# Bivalves are NO different: nitric oxide as negative regulator of metamorphosis in the Pacific oyster, *Crassostrea gigas*

**DOI:** 10.1186/s12861-020-00232-2

**Published:** 2020-11-23

**Authors:** Susanne Vogeler, Stefano Carboni, Xiaoxu Li, Nancy Nevejan, Sean J. Monaghan, Jacqueline H. Ireland, Alyssa Joyce

**Affiliations:** 1 Department of Marine Science, University of Gothenburg, Carl Skottbergsgata 22 B, 41319 Gothenburg, Sweden; 2 Institute of Aquaculture, University of Stirling, FK9 4LA Stirling, Scotland, UK; 3 South Australia Research and Development Institute Aquatic Sciences Centre, 2 Hamra Ave, West Beach, SA 5024 Australia; 4 Department of Animal Production, Ghent University, Coupure Links 653, 9000 Ghent, Belgium

**Keywords:** Nitric oxide, Nitric oxide synthase NOS, cGMP, Metamorphosis, Bivalves, *Crassostrea gigas*, Pacific oyster

## Abstract

**Background:**

Nitric oxide (NO) is presumed to be a regulator of metamorphosis in many invertebrate species, and although NO pathways have been comparatively well-investigated in gastropods, annelids and crustaceans, there has been very limited research on the effects of NO on metamorphosis in bivalve shellfish.

**Results:**

In this paper, we investigate the effects of NO pathway inhibitors and NO donors on metamorphosis induction in larvae of the Pacific oyster, *Crassostrea gigas.* The nitric oxides synthase (NOS) inhibitors s-methylisothiourea hemisulfate salt (SMIS), aminoguanidine hemisulfate salt (AGH) and 7-nitroindazole (7-NI) induced metamorphosis at 75, 76 and 83% respectively, and operating in a concentration-dependent manner. Additional induction of up to 54% resulted from exposures to 1H-[1,2,4]Oxadiazole[4,3-a]quinoxalin-1-one (ODQ), an inhibitor of soluble guanylyl cyclase, with which NO interacts to catalyse the synthesis of cyclic guanosine monophosphate (cGMP). Conversely, high concentrations of the NO donor sodium nitroprusside dihydrate in combination with metamorphosis inducers epinephrine, MK-801 or SMIS, significantly decreased metamorphosis, although a potential harmful effect of excessive NO unrelated to metamorphosis pathway cannot be excluded. Expression of *CgNOS* also decreased in larvae after metamorphosis regardless of the inducers used, but intensified again post-metamorphosis in spat. Fluorescent detection of NO in competent larvae with DAF-FM diacetate and localisation of the oyster nitric oxide synthase *CgNOS* expression by in-situ hybridisation showed that NO occurs primarily in two key larval structures, the velum and foot. cGMP was also detected in the foot using immunofluorescent assays, and is potentially involved in the foot’s smooth muscle relaxation.

**Conclusion:**

Together, these results suggest that the NO pathway acts as a negative regulator of metamorphosis in Pacific oyster larvae, and that NO reduction induces metamorphosis by inhibiting swimming or crawling behaviour, in conjunction with a cascade of additional neuroendocrine downstream responses.

**Supplementary Information:**

The online version contains supplementary material available at 10.1186/s12861-020-00232-2.

## Background

A biphasic lifecycle is common in many aquatic invertebrate species, including a planktonic larval stage for dispersal and a sessile adult stage, with metamorphosis marking the transition from one life stage to the other. In bivalve species such as oysters, metamorphosis occurs when swimming pediveliger larvae become ‘competent’, wherein they sink to the bottom and begin searching for an adequate surface to settle [1]. When environmental cues meet requirements for a suitable settlement surface, larvae undergo metamorphic changes, losing larval organs such as velum and foot that allow them to swim or crawl, and instead gain adult organs such as gills that are better adapted to a sessile lifestyle; thus, entering their juvenile stage, also known as spat. Complex neuroendocrine pathways and neurotransmitters have been proposed as regulators for metamorphosis in bivalves (reviewed in [2]), but there are many unknown factors regulating this process. Recently, we provided evidence for a previously unexplored regulatory pathway involved in bivalve metamorphosis involving the transmembrane *N*-Methyl-D-aspartate (NMDA) receptor [3–5]. As ligand-gated and voltage-dependent ion channels, NMDA receptors are located in synaptic cell membranes and are activated by agonists, predominantly glutamate, co-agonists (e.g. glycine) as well as the required dislodgement of a magnesium ion block from the inner ion pore by depolarisation of the cell membrane. The opening of the receptors causes an inflow of Ca^2+^ leading to an increase in the intracellular Ca^2+^ concentration, which in turn generates downstream responses such as activation of enzymes, gene regulation, or other cell-specific responses (reviewed in [6]). We demonstrated previously that exposure of competent larvae of different oyster and clam species to MK-801, a NMDA receptor specific channel blocker, significantly induced metamorphosis in these species [3]. Cloning and characterisation of NMDA receptors in the Pacific oyster *Crassostrea gigas* as well as localisation of NMDA receptor subunit 1, CgNR1, in key structures of competent larvae such as the apical sensory organ (ASO), the underlying apical/cerebral ganglia and the nerve network of the foot [5], established the existence of functional NMDA receptors in bivalve nervous systems. Both the ASO and the foot are structures specific to larval stages, that disappear after metamorphosis, which are assumed to be involved in sensing the environment for settlement cues [7–9]. The ASO, in particular, is an organ that is present from trochophore larval stage and persists in competent larvae until just prior to metamorphosis in most aquatic biphasic invertebrates. It is associated with sensory functions and combines an apical tuft with long cilia, sensory cells, and the apical and cerebral ganglia [10–12]. Based on the combined findings of our previous work, it is apparent that NMDA receptors are part of the regulatory mechanism of bivalve metamorphosis and more specifically, that opening of NMDA receptors initiates intracellular signalling and cells specific responses that negatively regulate metamorphosis.

In vertebrates, NMDA receptor downstream responses can be linked to the production of nitric oxide (NO) via a Ca^2+^/calmodulin pathway with NMDA receptors regulating the intracellular Ca^2+^ concentration. Calcium functions as a second messenger and binds to calmodulin, which subsequentially activates a nitric oxide synthase (NOS). The NOS is the key enzyme in the production of NO and catalyses L-arginine and NADPH to L-citrulline, NO, and NADP. Despite this, information on NO as a potential regulator of bivalve metamorphosis is limited. A recent 2020 study on the hard-shelled mussel, *Mytilus coruscus,* is the only known study in another bivalve that has shown NOS inhibitors induce metamorphosis, while NO donors inhibit the transformation [13]. Furthermore, the mussel’s NOS expression decreased in pediveliger larvae. This finding is not surprising given that NO has also been suggested as negative regulator of metamorphosis in a wide range of other biphasic invertebrates, including various gastropods [14–19], polychaetes [20], barnacles [21, 22], sea urchins [23, 24], nudibranchs [25] and ascidians [26, 27]. Inhibition of the NO pathway by exogenous NO scavengers or inhibitors to NOS has successfully induced metamorphosis in these species. However, interestingly for some gastropod, ascidian and sponge species, NO stimulated metamorphosis instead [28–32], suggesting some species-specific adaptation in response to NO [33]. Nevertheless, NO biosynthesis by NOS is a conserved pathway, found in all types of living organisms from prokaryotes, plants to metazoans with a remarkable conservation of animal NOS despite several duplication events in invertebrates and vertebrates [34–36]. Nitric oxide synthase expression and NO presence have been detected prior to metamorphosis in larval organs and body structures that are involved in sensing environmental settlement cues (e.g. mouth and ASO), such as NOS cells in the pharynx of annelid larvae [20], in the post oral ciliary band and oral ganglion in sea urchins [23, 24, 37], in the ASO and apical ganglion of the snail *Ilyanassa obsoleta* [17, 38, 39] and the foot of *Haliotis asinina* [28], or in structures that disappear during phase changes such as NO detection in tail regression of the ascidian *Ciona intestinalis* [27]. Expression of NOS also decreased in the snail *I. obsoleta* when metamorphosis was induced [40], while in the abalone *H. asinina*, one of the positively NO-regulated species, NOS expression increased after induction [28]. Several theories have emerged regarding NO downstream effects, inducing metamorphosis including a NO-regulated activation of soluble guanylyl cyclase (sGC) converting guanosine triphosphate (GTP) to cyclic guanosine monophosphate (cGMP), which can lead to further downstream responses within cGMP-gated ion channels, phosphodiesterases (PDEs), or protein kinases G (PKG) potentially inhibiting metamorphosis [16, 21, 23, 25, 41]. Another theory includes a negative regulation of apoptosis by NO [17], with apoptosis being an important process during metamorphosis leading to the loss of redundant larval organs. Furthermore, in species with NO-inducing effect, activation of the mitogen-activated protein kinase/extracellular-signal-regulated kinase (MAPK/ERK) pathway and subsequent regulation of metamorphosis-related genes have been suggested [30–32].

Participation of NO pathways in regulating metamorphosis, however, has not been investigated in many bivalve species, although functional NOS homologs and NO productions have been identified in the adult Pacific oyster, *C. gigas*, [42] and other bivalve species [13, 43–46]. Metamorphosis is a life stage transition characterised by very high mortality rates in bivalves, understanding how metamorphosis is regulated, and can quickly be induced, is important for several reasons. First, it is important to the survival of natural bivalve populations, as it allows for an understanding of the effects of environmental pollutants on key development stages, but as such, could also assist in the development of antifouling agents to prevent biofouling of unwanted bivalve species. It is also important to increase spat production in the aquaculture industry, where induction of metamorphosis leads to better survival as well as synchrony of cohorts during hatchery production. In this paper, we provide the first evidence that the NO pathway negatively regulates metamorphosis in the Pacific oyster, *C. gigas*. We exposed competent oyster larvae to various NOS inhibitors, to the sGC inhibitor 1H-[1,2,4]Oxadiazole[4,3-a]quinoxalin-1-one (ODQ) as well as to two NO donors in combination with known metamorphosis inducers. The gene expression profiles of *CgNOS* in veliger larvae and after metamorphosis induction were analysed. Detection of NO in competent larvae, localisation of *CgNOS* and *CgNR1* expression in competent and induced larvae and spat through in-situ hybridisation, as well as the localisation of cGMP by immunofluorescent assay in larval histological sections and whole-mount larvae, provides further information on the potential role of the NO pathway to regulate bivalve metamorphosis.

## Results

### Effect of NO pathway inhibitors and NO donors

**Fig. 1:**
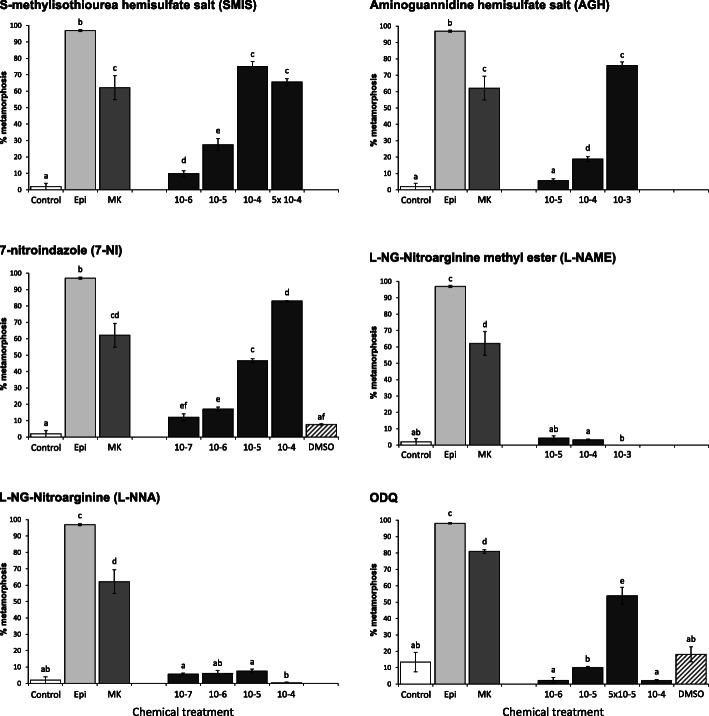
Percentage (%) of metamorphosis in Pacific oyster larvae after 24 h continuous exposure to single treatments (black bars) of NO pathways inhibitors SMIS, AGH, 7-NI, L-NAME, L-NNA and ODQ, at different concentrations, as well as known inducers epinephrine (EPI; light grey bars) and MK-801 (MK; grey bars) at 10^− 4^ M for 3 h, a DMSO (black-stripe bars) and a no treatment control (open bars). Data were collected 24 h post exposure start. *C. gigas* larvae from December 2018 experiment with competent larvae 18 dpf were used for all treatments except for ODQ, for which 19 dpf larvae were used. Error bars represent standard error. Different lower-case letters represent significant differences with *p* < 0.05

Effects on metamorphosis induction of the specific NOS inhibitors S-methylisothiourea hemisulfate (SMIS), aminoguanidine hemisulfate (AGH), 7-nitroindazole (7-NI), L-NG-nitroarginine methyl ester (L-NAME) L-NG-nitroarginine (L-NNA) and the specific sGC inhibitor ODQ were tested in 18 days post fertilisation (dpf) and 19 dpf competent Pacific oyster larvae, spawned in December 2018 (Fig. 1). Competence of larvae was verified by exposing larvae to epinephrine (EPI) and MK-801 for 3 h at 10^− 4^ M, with both compounds resulting in induction percentages of 96.9 ± 0.6% for EPI and 62.1 ± 7.3% for MK-801 in 18 dpf larvae, and 98.1 ± 0.4% for EPI and 81.0 ± 1.0% for MK-801 in 19 dpf larvae, respectively. Metamorphosis in the non-treatment control was low with 2.0 ± 2.0% when 18 dpf larvae used, but increased with 19 dpf larvae to 13.4 ± 6.0%. For both experimental sets, the DMSO controls did not significantly differ from the non-treatment control.

Compared to the non-treatment control, significant induction of metamorphosis was achieved in 18 dpf competent larvae after 24 h continuous exposure to SMIS, AGH and 7-NI at different concentrations with most effective concentrations for SMIS at 10^− 4^ M with 75.1 ± 3.1%, for AGH at 10^− 3^ M with 75.9 ± 2.1% and 7-NI at 10^− 4^ M with 83.0 ± 0.3% metamorphosis. The same larvae set was also exposed to two additional NOS inhibitors, L-NAME and L-NNA, but both compounds did not significantly induce metamorphosis. Similar results were obtained when 19 dpf larvae were used with significant induction of metamorphosis for SMIS, AGH and 7-NI and no induction for L-NAME and L-NNA (Additional file 1). The sGC inhibitor ODQ also induced metamorphosis with most effective concentration of 5 × 10^− 5^ M with 53.8 ± 5.2% in 19 dpf larvae. Metamorphosis induction was also achieved with ODQ in 18 dpf larvae, but metamorphosis induction was less effective and fewer concentrations without 5 × 10^− 5^ M was tested (Additional file 1).

During exposure and final assessment of larvae and spat, several noteworthy behaviour differences were observed between treatments. During EPI and MK-801 exposure larvae are generally immobile on the bottom, and spat induced with EPI are unattached, while MK-801 spat are partially attached and unattached as previously shown [3]. Some of those bottom laying larvae showed visible contractions of the larval organs – a behaviour often seen few hours after start of exposure to EPI, and might suggest larvae were undergoing metamorphosis. Spat, which metamorphosis was induced by exposing them to SMIS, AGH and 7-NI were predominantly attached to vial’s bottoms and sides. The appearance of spat after AGH treatment, in particular, were subjectively smaller with less adult shell growth compared to all other successful treatments. Interestingly, attachment of animals to the surface appeared early in those treatments. During sampling for the gene expression analysis, larvae exposed to SMIS, AGH and 7-NI were predominantly stuck to the surface (‘stickiness’) with those not-stuck either swimming or crawling. Larvae exposed to ODQ were not attaching to the bottom, and mostly and swimming or crawling instead. Only a small fraction of spat was attached, similar to MK-801 spat. L-NAME and L-NNA treated larvae were swimming and crawling throughout exposure and did not differ in their behaviour from the no-treatment control. DMSO control larvae were immobile on the bottom during exposure and spat were either attached or unattached.

**Fig. 2:**
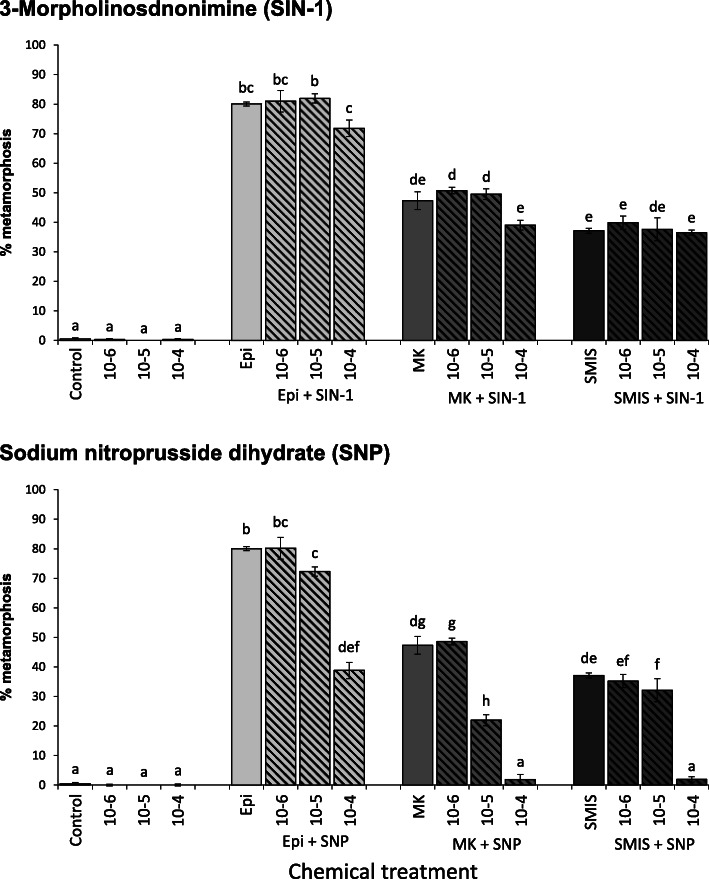
Percentage (%) of metamorphosis in Pacific oyster larvae after 24 h continuous exposure to single treatments (black bars) of NO donors SIN-1 and SNP at 10^− 6^ M to 10^− 4^ M or as co-exposures (hashed bars) with epinephrine (Epi, light grey bars), MK-801 (MK, grey bars) and SMIS (dark grey bars) at 10^− 4^ M for 3 h exposure followed by continuous single exposure to SIN-1 or SNP at 10^− 6^ M to 10^− 4^ M, and a non-treatment control. Data were collected 24 h post exposure start. *C. gigas* larvae from March 2019 experiment with competent larvae 18 days post fertilisation were used for all treatments. Error bars represent standard error. Different lower-case letters represent significant differences with *p* < 0.05

Effects of NO donors as metamorphosis inhibitor were tested in co-exposures of larvae (18 dpf, March 2019) with an inducer such as EPI, MK-801 or SMIS together with a potential inhibitor, the NO donor 3-morpholinosydnonimine (SIN-1) or sodium nitroprusside (SNP) for 3 h, followed by a continuous exposure to the NO donor alone (Fig. 2). The NO donor SNP significantly inhibited metamorphosis at concentrations of 10^− 5^ M and 10^− 4^ M, while the effect of SIN-1 did not significantly decrease metamorphosis percentages compared to inducers alone. Besides the reduction of spat in the high concentration treatments of SNP, spat exposed to SNP at 10^− 4^ M displayed limited adult shell growth compared to single inducer exposed spat and remaining larvae were immobile on the bottom.

**Fig. 3:**
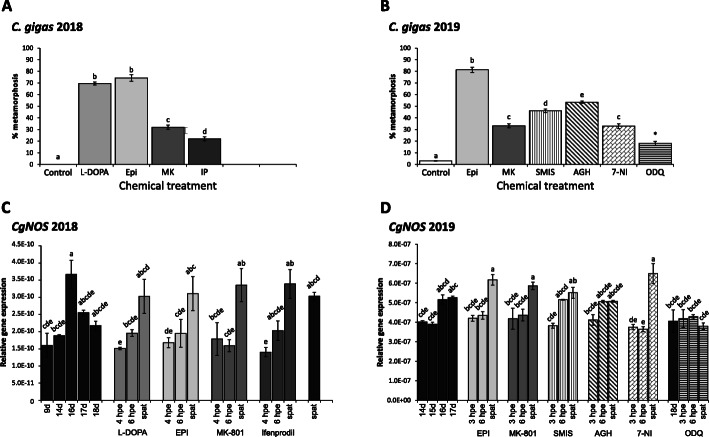
Gene expression of *CgNOS* in Pacific oysters at different larval stages, larvae exposed to different metamorphosis inducers and spat 24 h after metamorphosis induction as well as metamorphosis percentages (%) in Pacific oyster larvae after exposure to inducers. A & C) *C. gigas* larvae, 18 dpf (d) in January 2018 experiments, exposed to epinephrine (EPI) and MK-801 (MK) at 10^− 4^ M, L-DOPA at 10^− 5^ M and ifenprodil (IP) at 10^− 6^ M and sampled at 4 hpe and 6 hpe. B & D) *C. gigas* larvae, 17 dpf or 18 dpf for ODQ in March 2019 experiments, exposed to EPI, MK-801, SMIS and 7-NI at 10^− 4^ M, AGH at 10^− 3^ M and to ODQ at 5 × 10^− 5^ M and sampled 3 hpe and 6 hpe. S: spat, C: control spat. Error bars represent standard error. Different lower-case letters represent significant differences with *p* < 0.05; *: significance not calculated due to differences in sampling days

Larvae, exposed to metamorphosis inducers, and subsequent spat, were sampled for gene expression analysis of a NOS homolog in Pacific oyster larvae. Larvae exposed in January 2018 and March 2019 (Fig. 3A & B) were successfully induced with EPI and MK-801 as well as L-DOPA and ifenprodil (*C. gigas* 2018) and the NO pathway inhibitors SMIS, AGH, 7-NI and ODQ (*C. gigas* 2019). Comparison between larval batches in December 2018 and March 2019 indicates that the responses to NO pathway inducers were also cohort dependent, as we have previously suggested for the NMDA receptor related compounds, MK-801 and ifenprodil [3]. Experiments conducted in March 2019 displayed lower metamorphosis percentages relative to experiments using larvae from December 2018 (Fig. 1) for all inducers with EPI with 81.3 ± 2.2%, MK-801 with 33.1 ± 1.7%, SMIS with 46.1 ± 1.5%, AGH with 53.5 ± 0.9% and 7-NI with 32.9 ± 2.1% (Fig. 3B). Any age dependent effects based on the fact that 18 dpf larvae were used in December 2018, and 17 dpf larvae in March 2019, could be excluded given that 18 dpf March 2019 larvae were also tested (Additional File 2) and, although for some compounds a higher metamorphosis percentage was achieved (e.g. MK-801 with 47.3 ± 3.0% and 7-NI with 64.5 ± 1.8%), the metamorphosis induction was overall lower compared to December 2018 experiments. Unfortunately, exposure to ODQ did not significantly induce metamorphosis in 17 dpf March 2019 larvae (4.1 ± 1.0%) and 18 dpf exposed larvae/ spat were sampled for gene expression analysis with metamorphosis induction of 18.1 ± 1.7% (Fig. 3B). However, results from ODQ samples should be interpreted with caution given that spat appeared smaller in size compared to EPI or SMIS treated animals and the DMSO control resulted in insignificant comparable metamorphosis percentage with 25.1 ± 3.8% (Additional file 2). The effect of exposure duration of NO pathway inhibitors was also tested in March 2019, with larvae induced either for 3 h, 6 h or 24 h. However, no significant differences were observed based on duration of exposure for SMIS, AGH, 7-NI and ODQ exposed larvae (Additional file 2).

The specific gene translating CgNOS, the protein that is suggested to produce NO in Pacific oysters, was expressed at different late larval stages, during and after exposure to metamorphosis inducers in competent pediveliger larvae and in spat in both sample sets from 2018 and 2019 (Fig. 3C & D). In general, both experiments showed comparable gene expression patterns for *CgNOS*, independent of metamorphosis inducers or difference in early exposure sampling time. *CgNOS* gene expression increased in pediveliger larvae around 16 dpf, but decreased in older pediveliger larvae at 17 dpf in 2018 and 18 dpf in 2019. All inducers except ODQ affected *CgNOS* gene expression in a similar trend with a decrease in expression in 3 h post exposure start (hpe) and 4 hpe larvae, followed mostly by a non-significant weak expression increase in 6 hpe larvae. In spat, however, *CgNOS* gene expression increased significantly after exposures to different metamorphosis inducers, except for AGH. Naturally occurring control spat also increased their expression of *CgNOS* compared to 18 dpf competent larvae in 2018, suggesting that a rise in *CgNOS* transcription in spat is a natural process independent of inducers. In general, none of the inducers displayed a unique pattern, with all inducers leading to a comparable reduction of *CgNOS* gene expression during metamorphosis, independently of any differences in the percentages of larvae per treatment that were induced to successfully undergo metamorphosis. One exception is ODQ treated larvae and spat, which did not show any differences in expression pattern up to 18 dpf competent larvae. The reason for this is not clear, but might be due to a side effect of the DMSO solvent and/or due to the slow development based on the smaller size of ODQ spat. A DMSO side effect was not observed in 7-NI treated animals in larvae or spat appearance, but it cannot be excluded.

### Localisation of NO, NOS, NMDA receptor and cGMP

**Fig. 4:**
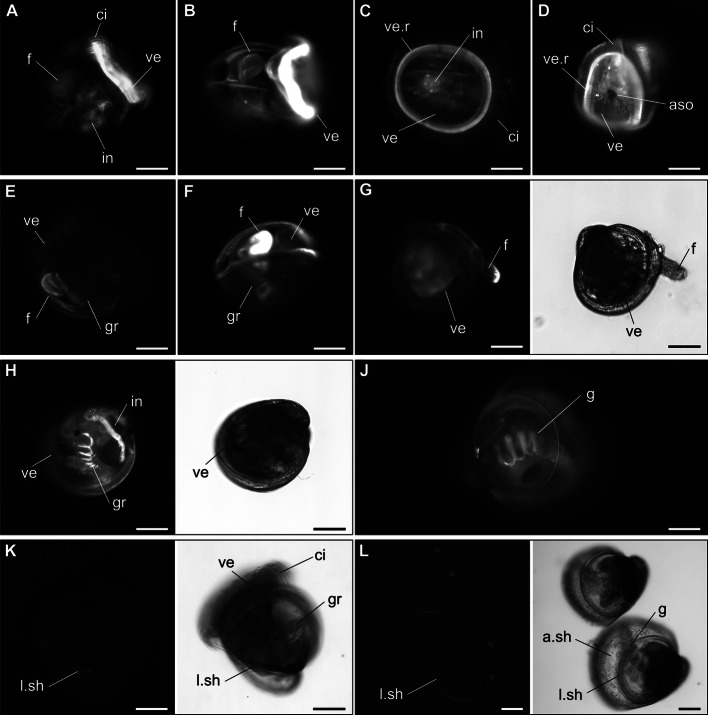
Nitric oxide (NO) detection in competent Pacific oyster larvae and spat using the NO-specific fluorescent indicator DAF-FM diacetate. Fluorescent and bright-field images of fluorescent NO signals in competent larvae swimming **a** left side view, **b** anterior view on foot, **c** ventral view on velum, **d** ventral view on velum with visible apical sensory organ; in larvae with predominant foot crawling on the bottom **e** left side view after AGH exposure, **f** anterior view and **g** left side view after MK-801 exposure; **h** larvae displaying organ contraction behaviour signalling metamorphosis after EPI exposure; **j** spat 24 hpe to EPI; control animals without DAF-FM diacetate treatment **k** in competent larvae without treatment and **l** spat hpe to EPI. aso: apical sensory organ, a.sh: adult shell growth, ci: cilia of the velum, f: foot, g: gills, gr: gill rudiments, l.sh: larval shell growth, ve: velum, ve.r: rim of velum. Scale bar: 100 μm

Nitric oxide was localised in competent larvae, in larvae exposed for 3 h to EPI, MK-801, SMIS, AGH and 7-NI, and in spat 24 hpe. Differences in NO position between exposed and unexposed competent larvae were not detected. However, differences in fluorescent NO signals were observed depending on larvae displaying particular behaviours such as swimming, crawling or laying immobile on the bottom while displaying an organ pulsing behaviour that suggests successful induction of metamorphosis (Fig. 4). In general, a NO signal was faintly visible in the intestine and rudimentary gills in most larvae independent of behaviour. In swimming larvae, NO signals were strongly detected in the velum (Fig. 4a & b), particularly on the rim of the velum (Fig. 4c & d). The foot and the apical tuft including the underlying ASO and ganglia region did not display noticeable NO signals (Fig. 4a & c), even when larvae were swimming with extended foot (Fig. 4b). The NO signal in the velum slowly faded when larvae stopped swimming and rested on the bottom. In crawling larvae actively searching the substrate, on the other hand, detectable concentrations of NO were predominantly in the foot, either the whole foot (Fig. 4e & f) or only the tip (Fig. 4g). Larvae that were displaying behaviour characteristic of successful induction, such as laying immobile on the bottom with visible contraction of larval organs, did not contain detectable NO concentration in the velum or foot, but NO was present in rudimentary gills and the intestine (Fig. 4h). In spat, a NO signal was only visibly present in the adult gills (Fig. 4j). Auto-fluorescent signals were not observed in animals not exposed to DAF-FM diacetate (Fig. 4k & l) with only a faint signal coming from the rim of the larval shell.

**Fig. 5:**
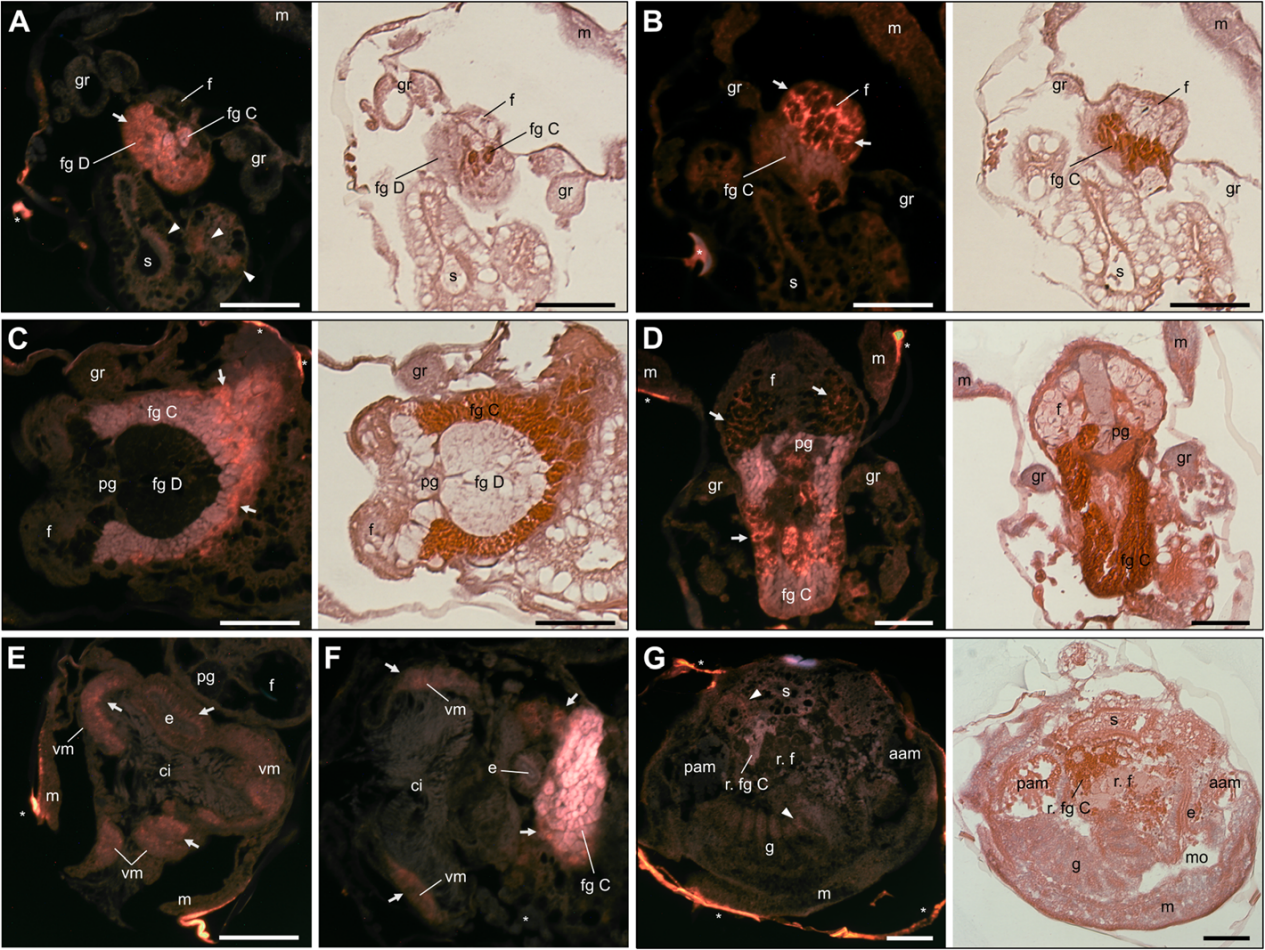
*CgNOS* (**a**, **c**, **e**-**g**) and *CgNR1* (**b**, **d**) expression localisation in competent Pacific oyster larvae and spat by in-situ hybridisation using digoxigenin labelled riboprobes (orange staining) with fluorescent signals visualised using a triple-band DAPI-FITC-Texas Red excitation filter. Frontal serial sections of foot area of the same larva 6 hpe to EPI with (**a**) *CgNOS* and (**b**) *CgNR1*, both with subsequent H&E staining of sections. Frontal sections of larvae (**c**) untreated with CgNOS and subsequent H&E staining of section, and (**d**) 6 hpe of EPI with *CgNR1* and H&E staining of consecutive section. Transverse section with *CgNOS* probe of competent larvae (**e**) untreated and (**f**) 6 hpe EPI treatment. (**g**) Sagittal section whole spat and H&E staining of consecutive section. White arrows and arrow heads: signal for successful probe binding. aam: anterior adductor muscle; ci: cilia of the velum; e: oesophagus; f: foot; fg C: foot glands C; fg D: foot glands D; g: gills; gr: gill rudiments; m: mantle; mo: mouth; pam: posterior adductor muscle; pg: pedal ganglia; r. f: remnants foot; r. fg C: remnants foot glands C; vm: vellum membrane; *: Fast red dye unspecific binding mostly in remains of periostracum. Scale bar: 50 μm

Localisation of *CgNOS* gene expression by in-situ hybridisation revealed a similar pattern as for the NO detection in competent larvae. *CgNOS* is not differentially expressed in untreated and treated larvae 3 hpe or 6 hpe to EPI, but rather generally expressed in the two key larval organs, the foot and the velum (Fig. 5). Transcripts of *CgNOS* in the larval foot are detected towards the base of the foot near the foot glands C, which seem to be covered and/or partially penetrated (Fig. 5a & c). The foot glands C together with foot glands D are presumed to be involved in the production and secretion of the adhesive that oysters produce to cement to the surface during settlement [47–49]. *CgNOS* expression, however, with its specific localisation near the foot base differs from *CgNR1*, the gene that expresses NMDA receptor subunit 1 CgNR1 and which is expressed in the nerve network penetrating the whole foot starting from the base of the foot towards the ciliated tip (Fig. 5b & d) [5]. Furthermore, *CgNOS* is not expressed in or near the apical/cerebral ganglia, as it has been shown previously for *CgNR1* [5]. Instead, *CgNOS* expression is located in the velum membrane (Fig. 5e & f), which after unfolding, forms the velum including a cilia band. Interestingly, strong accumulation of *CgNOS* expression were not detected in spat 24 hpe of EPI (Fig. 5g), although *CgNOS* displayed higher expression levels in spat compared to larvae treated with any of the metamorphosis inducers (Fig. 3). Only faint signals were detected in the gills and in structures in the visceral mass (white arrow heads, Fig. 5g) as seen for larvae exposed to EPI (Fig. 5a). Furthermore, no signals were detected in areas of the spat section of what appears to be remnants of the larval foot. Even though predominant adult shell growth as well as adult gills are clearly visible in 24 h spat, often remnants of the foot and occasionally velum are still present, which have not yet been completely reabsorbed by the animal after 24 h post metamorphosis. Complete loss of larval organs can take 1 to 3 days depending on bivalve species [50–53]. Fluorescent signals were also detected along the pericostracum and occasionally on the edges of the animals (stars Fig. 5). These signals are relics of the Fast Red staining and were also observed in sections incubated with the sense probe for *CgNOS* (non-specific binding control), which did not display any signs for the unspecific probe or Fast Red binding elsewhere in the larval tissues sections (Additional file 3). No staining was observed in the negative controls.

**Fig. 6:**
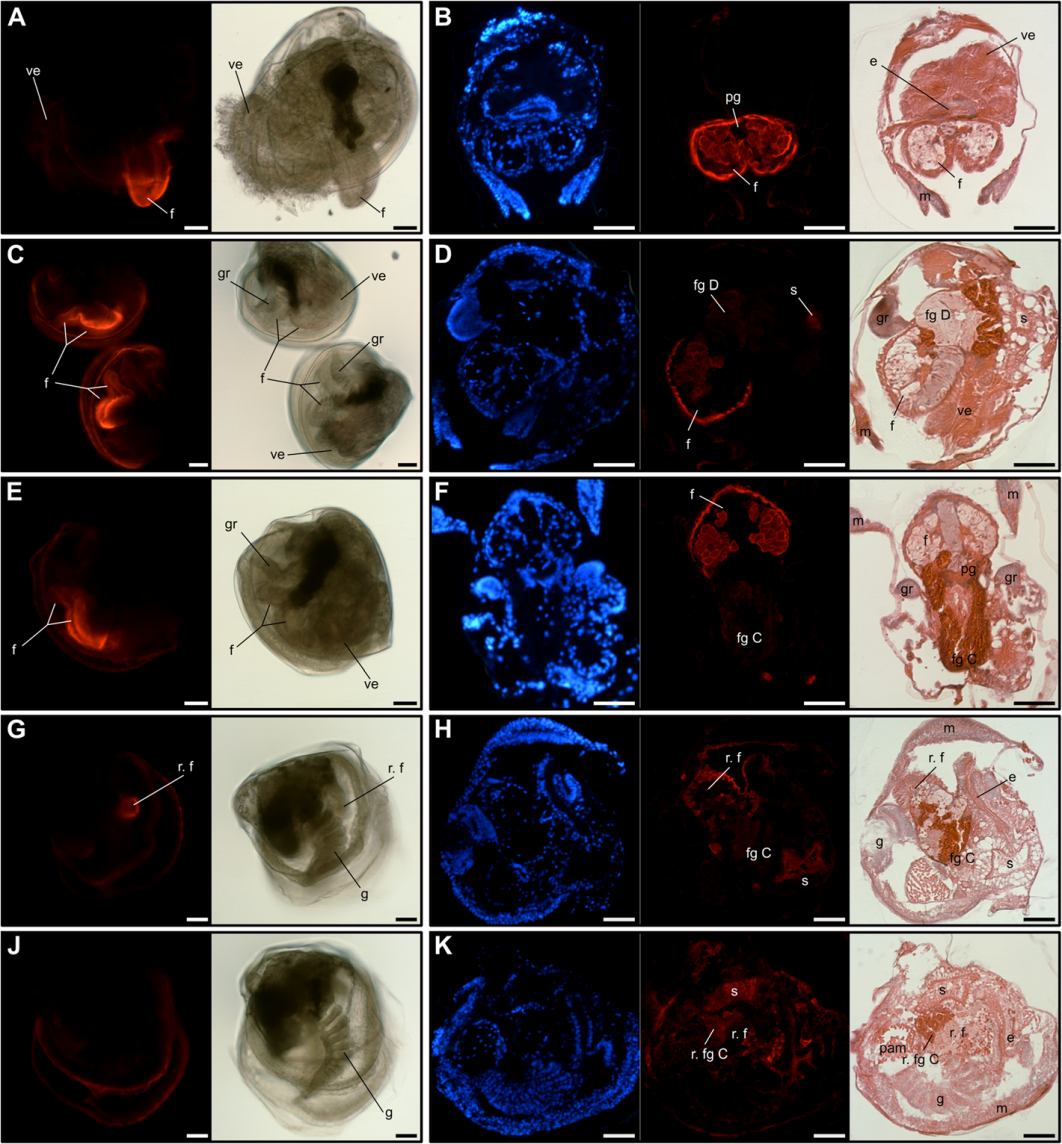
cGMP immunostaining in Pacific oyster larvae (**a**-**f**) and spat (**g**-**k**). Fluorescent and bright-field images of whole-mount stained individuals: 16 dpf larva (A, flattened with cover slip), 17 dpf larvae (**c**), 17 dpf larva 3 hpe EPI (**e**), spat with visible foot remnants (**g**), spat without visible foot remnants (**j**); fluorescent images of sectioned individuals accompanied with superimposed DAPI/cGMP signals as well as H&E staining: transverse section 17 dpf larva (**b**), sagittal section 17 dpf larva (**d**), frontal section 17 dpf larva 6hpe EPI (**f**), sagittal sections spat with large (**h**) and small foot remnants (**k**). e: oesophagus; f: foot; fg C: foot glands C; fg D: foot glands D; g: gills; gr: gill rudiments; m: mantle; pam: posterior adductor muscle; pg: pedal ganglia; r. f: remnants foot; r. fg C: remnants foot glands C; ve: velum. Scale bar: 50 μm

Spatial distribution of cGMP in larvae and spat by immunofluorescent labelling shows cGMP immunoreactivity in the foot of oyster larvae in whole-mount staining (Fig. 6a, c, e) and tissue sections (Fig. 6b, d, f). The larvae sections specifically show staining in ciliated foot including the outer cell walls and in cell walls of large individual cells within the foot tip. No signals were detected in the pedal ganglia, foot glands C or D. Cyclic guanosine monophosphate immunoreactivity in the foot tip was generally observed across all larvae samples from 14 dpf to 17 dpf larvae 6 hpe EPI treatment (Fig. 6a-f, Additional file 4). Positive signals were also observed in spat when large foot remnants were visible (Fig. 6g & h), but were absent when no visible foot remnants were detected in whole-mount stained spat, or confined to a very small foot remnant without foot tip in spat sagittal sections (Fig. 6j & k). Additional weak signals were detected around the stomach in sections of larvae (Fig. 6d) and spat (Fig. 6h & k). Negative controls and non-specific binding controls did not show significant immunoreactivity in larvae or spat near the ciliated foot area during whole-mount staining or in sections (Additional file 5). Western blot analysis confirmed anti-cGMP polyclonal antibody (PAb) binding to oyster larvae protein extracts, while no band was detected for the negative control or for the anti-uNOS PAb, for which no successful immunoreactivity during immunofluorescent assay were obtained (Additional file 5).

## Discussion

Successful induction of metamorphosis in Pacific oyster larvae was achieved after exposure to the either of the three NOS inhibitors SMIS, AGH or 7-NI, or to the sGC inhibitor ODQ, providing the first evidence that the NO pathway acts to inhibit metamorphosis in the Pacific oyster, *C. gigas*. The inducing effect of these NO pathway inhibitors, however, varies between larval cohorts, thus confirming that the internal NO concentration is not the only factor regulating metamorphosis. In addition to batch-related variability, which has also been observed previously for other inducers of Pacific oysters [3, 5] and other bivalve species [2], reported differences in effectiveness of the NOS inhibitors might also be caused by the binding specificity of each compound to different NOS isozymes. SMIS is a relatively specific inhibitor of vertebrate inducible NOS (NOS II), while AGH is an inhibitor of NOS II as well as vertebrate neuronal NOS (NOS I), and 7-NI is advertised to inhibit NOS I, although its binding selectivity has been questioned [54]. Similar to many other invertebrates, the Pacific oyster possesses one NOS homolog CgNOS [42], without a linage specific duplication that occurs occasionally in invertebrates, and is evidenced in several other molluscan species [55–57]. While CgNOS displays its highest sequence identity and structural similarities with vertebrate NOS I [42], it is not a direct homolog to either one of the two vertebrate NOSs or a third endothelia NOS (NOS III). These three NOS homologs derived from vertebrate-specific gene duplications [34–36]. Therefore, binding ability might vary, and could also explain the ineffectiveness in inducing metamorphosis of some NOS inhibitors such as the inability of L-NAME, a vertebrate non-selective NOS inhibitor, and L-NNA, selective to NOS I and NOS III. While both compounds have been successfully used to induce metamorphosis in other invertebrates [18, 23, 25, 27], L-NAME in particular did not significantly decrease the NOS activity in the scallop *C. farreri* while SMIS and spermidine trihydrochloride, another NOS I inhibitor, reduced its activity [43]. L-NAME also did not induce metamorphosis in annelids even at high concentrations [20]. However, L-NAME induced metamorphosis in the hard-shelled mussel [13] suggesting that inability to react to L-NAME might not be a general bivalve trait. Further research is needed to predict the binding ability of vertebrate NOS inhibitors to CgNOS. It can also not be excluded that L-NAME and L-NNA are not sufficiently taken up by larvae and/or transported to the site of action in our experiments given that bath applications with L-NAME in the slipper snail *Crepidula fornicata* did not induce metamorphosis [14, 58], while injection in the marine snail *I. obsoleta* had an inducing effect [15, 16].

Our results further demonstrated that metamorphosis induction was inhibited by the NO donor SNP after larvae were induced with compounds associated with potential regulatory pathways, including the adrenergic pathway with EPI, the NMDA receptor pathway with MK-801 and an NOS pathway with SMIS as NOS inhibitor. This could suggest that the NO pathway is required once an adrenergic or NMDA receptor pathway is induced. However, this theory has to be evaluated with caution. High concentrations of SNP were required to inhibit metamorphosis. Therefore, a harmful effect of excessive NO cannot be excluded given that larvae were immobile on the bottom of the vials and spat developed poorly. Nitric oxide has been shown to have a wide range of biological functions in marine invertebrates related to feeding, learning, defence and immune responses, as well as to environmental stress [59]. Nitric oxide also inhibits gill respirations in adult bivalves [60, 61] and insufficient oxygen supply could potentially provide an unfavourable environment for metamorphosis independent of the larva’s own internal NO-regulated pathways. Inhibition of metamorphosis in other invertebrates has been mostly observed at high SNP concentration similar or higher to the concentration used in this study [20–22]. Metamorphosis in the ascidian *H. momus*, a positive NO-regulated species, was inhibited by high concentrations of S-nitroso-N-acetyl-penicillamine (SNAP), another commonly used NO donor, although lower concentration significantly induced metamorphosis [29], supporting a theory of a potentially negative impact of excessive NO in bath applications unrelated to regulatory pathways involved in metamorphosis. Interestingly, the induction effect of AGH on metamorphosis in *M. corcurus* mussels was significantly inhibited by a 15 min exposure to two NO donors, SNAP and SNP, and to L-arginine prior to the 24 h exposure to AGH [13]. The authors concluded that exposure to exogeneous NO can supress larval metamorphosis. However, a conclusive explanation on how such short exposure to NO donor or L-arginine can inhibit metamorphosis for longer than 24 h, when an effective inducer is present, is still required.

Nevertheless, a reduction of NO production seems to be required for successful induction and execution of metamorphosis in *C. gigas*. This is supported by a decrease in *CgNOS* expression after induction of metamorphosis regardless of the inducer type. Decrease in *NOS* expression during metamorphosis seems common in invertebrate species whose metamorphosis is inhibited by NO [27, 40], while upregulation of the *NOS* gene has been reported in species which require NO for metamorphosis [29]. In the nudibranch *Phestilla sibogae*, a species for which metamorphosis can be induced by NO pathway inhibitors, NO production significantly decreased after induction of metamorphosis [25]. Yet, for the slipper snail *C. fornicata* and the abalone *H. asinina,* fluctuations of *NOS* expression during metamorphosis have been described, suggesting that *NOS* expression and subsequent NO production is not just either up or downregulated throughout metamorphosis, but might vary depending on metamorphosis progress, and could also be affected by experimental conditions (e.g. handling stress, different rearing conditions) [28, 62], which might also explain the minor differences in *CgNOS* expression between the inducers.

Reduction in *CgNOS* expression during metamorphosis is likely to be related to its spatial expression. *CgNOS* is predominately expressed in the velum membrane as well as at the base of the foot, the two larval organs that are lost during metamorphosis, which consequentially leads to a decrease in *CgNOS* expression during this process. However, both organs are crucial during the larval life stage. As noted, *CgNOS* expression increased during late larval development, at which time the velum is growing and the foot developing (approx. 14–16 dpf). Increase in NOS expression has also been reported in scallop *C. farreri* larvae with maximum expression in the late larval stage [43], but unfortunately, no information was provided about *CfNOS* during metamorphosis. In the hard-shelled mussel, *NOS* expression decreased in pediveliger compared to the earlier larval stages, while the NOS activity in pediveliger increased [13], suggesting that low expression does not always correlate with low activity. Our results further show that NO was present in the velum when oyster larvae were swimming, and absent in the velum when resting on the bottom. Crawling larvae displayed high NO concentrations in the foot, but NO was not detected in the velum or the foot when larvae displayed typical organ pulsing behaviour, thus suggesting successful induction of metamorphosis. Inhibition of NOS and subsequent reduction of NO by NOS inhibitors might therefore partially relate to an impact on the swimming and crawling ability of larvae, potentially signalling as substitution of a specific environmental cue or imitating internal signals for adequate conditions to initiate metamorphosis. Given that metamorphosis was not induced in all larvae after exposure to NOS inhibitors, additional prerequisite conditions and pathways seem to be required to induce metamorphosis. Interestingly, expression of *CgNOS* increased again in spat without strong spatial expression. The DAF-FM diacetate assay localised NO predominately in the gill. Faint signals of *CgNOS* expression were also detected in the gills and in the visceral mass including the stomach and the intestine. Nitric oxide synthase and NO-ergic cells have previously been reported in gills, digestive gland and intestine of adult bivalves [63–65], suggesting the main function of NO in spat is around respiration and digestion.

Upstream regulation and activation of NOS and the NO pathway in Pacific oyster larvae are likely to be regulated by Ca^2+^/calmodulin similar to vertebrate NOS [54] as previously suggested for gastropod species [66, 67]. Moreover, intracellular Ca^2+^ concentrations might be regulated by NMDA receptors. In adult snails *L. stagnalis*, an increase in NO production was achieved after exposure to NMDA and glutamate, two NMDA receptor agonists, and reversed by the NMDA receptor channel blocker MK-801 [68]. Our study showed that in *C. gigas* larvae *CgNOS* as well as *CgNR1* are both expressed in the foot, particularly near the foot base, supporting this theory of NMDA receptor regulating NO production via intracellular Ca^2+^ concentrations and subsequent activation of CgNOS. Co-localisation of NMDA receptors and NOS in vertebrates mostly occur on the postsynaptic side or neuromuscular junctions [69]. Thus, CgNOS could receive signals from NMDA receptors for further downstream pathways potentially involved in regulating foot glands for cementation [47–49] or muscle fibres ([69], and references herein), both also located at the base of the foot [47, 70]. Physical interaction between vertebrate transmembrane NMDA receptors and NOS, when not soluble in the cytosol, also exists via postsynaptic density proteins 95 (PSD-95), which binds to the PDZ domains of the NMDA receptor and NOS [71]. CgNOS have been shown to successfully interact with CgPSD-95 [42] and *C. gigas* NMDA receptor sequence analysis indicated PDZ domains [5]. The extended network of NMDA receptor expressing cells from the base, including foot glands D, and into the foot (this study and [5]) might take part in forwarding internal signals or responses to exogenous environmental cues received from the tip to base of the foot. The foot of pediveliger larvae have long been suggested as sensor of environmental cues [8, 9]. In contrast, the ASO, the main larval organ suspected of sensing environmental cues, did not express *CgNOS,* and NO was not detected near the apical tuft or the underlying apical ganglia, although our previous work has shown that the *CgNR1* is expressed in the apical/cerebral ganglia area [5]. Therefore, CgNOS does not seem to be directly involved in responding to environmental cues sensed by the ASO through an NMDA receptor cascade. However, production of NO in the velum membrane by CgNOS could still be regulated by the ASO and underlying ganglia. In addition to NMDA receptor presence, the apical/cerebral ganglia complex consists of several serotonergic, acetylcholinergic and FMRFamide reactive neurons, from which in particular serotonin and acetylcholine neurites innervate the ciliated velum [72], potentially interacting with CgNOS and NO in the velum membrane and rim. Both neurotransmitters have been previously linked to metamorphosis regulation in several bivalve species [2] and other biphasic invertebrates [16, 19, 20, 73].

The cGMP presence in the foot concurred with NO detection in the foot of crawling larvae as well as with the NOS localised at the base of the foot, suggesting that cGMP is part of the downstream NO pathway. Synthesised NO at the base of the foot by NOS, which is transported or diffuses into the foot, activates the sGC to produce cGMP. Nitric oxide activating sGC and raising intracellular cGMP concentration has also been suggested for other invertebrate larvae [14, 23–25, 74]. The presence of cGMP in all larval stages that have a developed foot further suggests that cGMP fulfils a more generic function than exclusively being involved in regulating metamorphosis induction. The cGMP interacts with PKGs, which are involved in phosphorylation of proteins leading to smooth muscle relaxation, with cGMP present at basal levels, either free or bound to PKGs or PDEs, the enzymes responsible for cGMP degradation (reviewed in [75]). Investigations of myogenesis in scallop *Nodipecten nodosus* larvae have shown that the foot muscles consist of bundles of striated and smooth muscle extending deep into the foot with foot retractor muscles predominately made from smooth muscles [76]. Thus, NO in *C. gigas* larvae might take part in controlling the foot movements through contraction and relaxation of smooth muscles, with contractions induced by calcium-regulated phosphorylation and relaxation regulated by cGMP, PKG and PDEs. Therefore, potential environmental cues that stimulate or inhibit endogenous NO production could regulate metamorphosis by keeping larvae either crawling or by impairing foot movement, thereby signalling an adequate settling surface. Regulation of smooth muscles by an NO/cGMP pathway probably also occurs in the stomach and digestive tract, which both have been shown to contain smooth muscle tissue in bivalve larvae and juveniles [76, 77] and our results indicate a weak cGMP presence in larvae as well as spat.

In contrast to the foot, cGMP was not detected in the velum, although NO was detected in the velum of swimming larvae, and *CgNOS* expressed in the velum membrane. It is possible that NO-regulated movement of the velum is not regulated by a NO/cGMP-pathway. This theory is supported by studies on the velum retractor muscles, which have been shown to be striated muscles in larvae of the oyster *C. gigas* [70], mussels [77] and scallops [76] with striated muscles being not regulated by cGMP. Thus, the effect of NO on velum locomotion might be implemented by an unknown effect on the cilia movement given that NO is predominantly present in the velum rim where the cilia are located. Differences in muscle types, and the regulating pathways involved in velum and foot movements, might also partially explain the observed differences in induction success of the three NOS inhibitors compared to ODQ, an sGC inhibitor and the least effective inducer of oyster metamorphosis in this study. ODQ inhibits the production of cGMP, consequently movement of the foot is impaired, but the velum is not affected. If a resting state in both organs is required for successful induction of metamorphosis, ODQ would only induce larvae whose velum had also received a metamorphosis-inducing signal. In contrast, signals caused by the three NOS inhibitors are directed to both the velum and the foot. The fact that spat produced as a result of ODQ exposure were unattached, also indicates that cGMP is not involved in the cementation process of the Pacific oyster. The fact that ODQ produces mostly unattached spat while the three NOS inhibitors produces mainly attached larvae and spat suggests instead that attachment of larvae is regulated downstream of the NOS and subsequent NO production, but independent of the NO/cGMP pathway. It is not clear yet how neuroendocrine pathways regulate the cementation of oysters during settlement. Exposure experiments with various neuro-active compounds either produce attached or unattached spat; for instance, EPI produces unattached spat, while L-DOPA causes attachment (Bonar et al., [9]; Shpigel et al., [78]). However, further research is needed to elaborate on how the NO pathway is potentially involved in cementation.

The effect of NO on oyster larvae seems to be focused on locomotion of larvae, with the presence of NO essential for swimming and crawling, while NO inhibition acts as an inducer for metamorphosis. However, NO pathways might regulate additional downstream responses, which should also be considered. Nitric oxide is an inhibitor of caspases [79], enzymes involved in regulating and executing apoptosis [80]. Programmed cell death, of which apoptosis is a form, has been detected in the apical ganglion of the snail *I. obsoleta* after metamorphosis induction by NOS inhibitors and serotonin [19]. In *C. intestinalis*, NO and caspase-3 regulate tail regression, with inhibition of NO production resulting in an increase in caspase-3 activity and acceleration of tail resorption, while an increase in NO levels causes a delay in this process [27]. Increases in caspase expression have been reported during Pacific oyster metamorphosis [81], and were localised in the velum and foot of Fujian oyster, *Crassostrea angulata*, larvae after induction to EPI [82]. The foot and velum are the two organs which in competent *C. gigas* pediveliger larvae express *CgNOS* and contain NO, as shown in our study. However, NO downstream regulation of apoptosis varies depending on concentration as well as cell type, given that NO can have opposing effects as a pro- and anti-apoptotic effector [83]. NO-regulated apoptosis might also involve several intermediate pathways including MAPK/ERK and c-Jun NH2-terminal kinase JNK pathways as suggested for ascidians and other biphasic invertebrates [30–32, 84–86]. Additional research is needed to understand if and how NO is involved in the apoptotic process wherein the velum and foot are lost during oyster metamorphosis.

In conclusion, NO is an essential negative regulator of metamorphosis in the Pacific oyster with an apparent effect on the locomotive ability of larvae. Based on our findings, we propose that NO detains larvae in the larval stage by promoting a swimming and crawling behaviour. When endogenous NO concentration decrease, either by an internal signal or as a response to environmental cues, swimming and crawling is impaired and larvae proceed with metamorphosis. However, the inability of NO pathway inhibitors to induce metamorphosis in all larvae suggests that other currently unknown pathways are also implicated. In addition to motility effects, other effects of NO are possible and might include the involvement of NO in regulating apoptosis, as well as the cementation process. Nevertheless, our data provide valuable new information about oyster metamorphosis and emphasize the importance of NO pathways during this key developmental stage of a bivalve species.

## Methods

### Chemical reagents

The catecholamine epinephrine hydrochloride (EPI), the NOS inhibitors S-methylisothiourea hemisulfate salt (SMIS) and aminoguanidine hemisulfate salt (AGH) as well as the NO donor sodium nitroprusside dihydrate (SNP) were purchased from Sigma-Aldrich. The irreversible inhibitor of soluble guanylyl cyclase 1H-[1,2,4]Oxadiazole[4,3-a]quinoxalin-1-one (ODQ) and the NO donor 3-morpholinosydnonimine chloride (SIN-1) were obtained from AdipoGen Life Sciences and the NOS inhibitors L-NG-nitroarginine methyl ester (L-NAME) hydrochloride, L-NG-nitroarginine (L-NNA) and 7-nitroindazole (7-NI) were purchased from Cayman chemicals. Additional chemicals were Levodopa (L-DOPA), obtained from Santa Cruz Biotechnology, as well as (+)-MK 801 maleate (MK-801) and Ifenprodil (+)-tartrate salt (ifenprodil), both obtained from Selleckchem. Stock solutions at 10^− 1^ M for SMIS, AGH and SNP or at 10^− 2^ M for remaining compounds were either prepared with autoclaved Milli-Q dH_2_O or with DMSO (Sigma-Aldrich) for 7-NI and ODQ. Working solutions (10x concentrate of final concentration of treatment) for each compound were prepared prior to experiments with 1 μm filtered fresh seawater (FSW).

### Animal husbandry and metamorphosis assay

Pacific oysters, *C. gigas,* were cultured at the South Australian Research and Development Institute in Adelaide, South Australia with larvae derived from several independent spawning events were used for experiments. Larvae from nine family lines were obtained from the selective breeding program in January 2018 (*C. gigas* 2018) and are described in [5]; larvae from six females and nine males were similarly obtained 12 months later in December 2018, and finally larvae from 19 family lines in March 2019 (*C. gigas* 2019). All larvae were reared in FSW, maintained at 24.5 ± 0.5 °C with a salinity of 36.5 ± 0.5 ppt and fed with a microalgal mixture of *Tisochrysis lutea*, *Pavlova lutheri*, *Chaetoceros calcitrans* and *Chaetoceros muelleri*.

All metamorphosis assays were conducted following a general protocol as previously described [3–5]. In brief, competent pediveliger larvae (shell length 300–330 μm, visible eyespot, crawling behaviour) were placed in FSW in glass shell vials using a pipet with a large tip opening, continuously exposed to chemical compounds for either 1 h, 3 h, 6 h or 24 h; the chemicals were then removed by pipetting, and 10 ml FSW including microalgae was added to each vial. For the 24 h continuous exposures, the microalgal mixture was added to the vials at the beginning of exposures as a proportion of the total volume of 10 ml in the vial. Differences in total FSW volumes during exposure were necessary in order to provide sufficient water volume to maintain an appropriate environment for the duration of exposure, while keeping chemical usage, costs and chemical waste to a minimum. Final chemical concentrations for exposure experiments for EPI and L-DOPA were based on Bonar et al. [9] and for MK-801 and ifenprodil based on Vogeler et al. [3, 4]. For compounds previously not used on *C. gigas* larvae, a dose-range was used at which most known settlement inducers for bivalves are operating. Controls, including a no-treatment control, as well as a DMSO control with maximum volume of DMSO equal to the highest volume solvent in working solutions were used. After 24 h, animals were assessed under an inverted microscope and early spat, live and dead larvae were counted. Mortality percentages varied from 0 to 7.6% in individual vials after 24 h, but did not differ significantly between treatments.

NO inhibitor and donor experiments - (A) Single exposures: approximately 80–115 larvae (December 2018; 18 dpf & 19 dpf) were exposed to EPI and MK-801 at 10^− 4^ M for 3 h in a total volume of 2.5 ml, and to SMIS, AGH, 7-NI, ODQ, L-NAME and L-NNA for 24 h continuously at final concentrations ranging from 10^− 7^ M to 10^− 3^ M. (B) Co-exposures: approximately 90–120 larvae (March 2019; 18 dpf) were exposed to single treatments of EPI, MK-801 and SMIS at 10^− 4^ M for 3 h in a volume of 2.5 ml, and to SIN-1 and SNP at 10^− 6^ M to 10^− 4^ M for 24 h continuously, as well as to co-treatments with either EPI, MK-801 or SMIS at 10^− 4^ M together with either SNP or SIN-1 at 10^− 6^ M to 10^− 4^ M for 3 h in a volume of 2.5 ml with a subsequent single exposure to either SNP or SIN-1 for the remaining 21 h continuously in a total volume of 10 ml.

NOS gene expression experiments – (A) *C. gigas* 2018: approximately 250–320 larvae (January 2018, 18 dpf) were exposed to EPI at 10^− 4^ M for 1 h, and to MK-801 at 10^− 4^ M, L-DOPA at 10^− 5^ M and ifenprodil at 10^− 6^ M for 3 h in a total volume of 2.5 ml as previously described in [5]. The larvae were either sampled at 4 hpe and 6 hpe as well as spat 24 hpe for further analysis, or kept in the vials for 24 h and assessed under an inverted microscope. (B) *C. gigas* 2019: approximately 250–320 larvae (March 2019, 17 dpf & 18 dpf) were exposed to EPI and MK-801 at 10^− 4^ M for 3 h in a volume of 2.5 ml, as well as to SMIS and 7-NI at 10^− 4^ M, AGH at 10^− 3^ M and ODQ at 5 × 10^− 5^ M for either 3 h or 6 h in a total volume of 5 ml, or for 24 h continuously. Larvae were either sampled at 3 hpe, 6 hpe or as spat 24 hpe or kept for 24 h assessment of metamorphosis. Larvae and spat attached to glass vials were gently detached with a sharp spatula. Three separate samples were taken at each sampling point with each sample consisting of three biological replicates.

### Gene expression analysis

*C. gigas* larvae of different developmental larval stages, larvae after exposure to metamorphosis inducers, as well as spat were analysed for the gene expression study: *C. gigas* 2018 animals at mid-veliger (9 dpf), late veliger (14 dpf), pediveliger larvae (16 dpf), competent pediveliger larvae (17 dpf & 18 dpf), larvae exposed to EPI, L-DOPA, MK-801 and ifenprodil at 4 hpe, 6 hpe and spat 24 hpe, were preserved in RNAlater (Sigma-Aldrich); *C. gigas* 2019 animals at 14 dpf − 18 dpf, larvae exposed to SMIS, 7-NI, AGH and ODQ at 3 hpe, 6 hpe and spat at 24 hpe, were preserved in PaxGene Tissue system (PreAnalytix).

An NOS homolog, *CgNOS* (GenBank ID: XM_011421861), was identified in the Pacific oyster genome (ASM29789v2, Annotation Release 101 [87]) by a tBLASTn search using protein sequences to human nNOS (GenBank ID: AAA36376), eNOS (GenBank ID: AAA36364) and iNOS (GenBank ID: AAA59171). A primer pair for *CgNOS* with an amplicon length of 185 bp was designed with Primer Blast at NCBI [88]: forward primer 5′-GAAGATGACCTCGGAGCAGG-3′, reverse primer 5′-TGACCACTTCATCAGTCCGC-3′. Relative gene expression for *CgNOS* was assessed for all samples using quantitative PCR based on a modified comparative Ct model [89] following the protocol as previously described with the elongation factor-1 α, ribosomal protein S18, ribosomal protein L7 as reference genes [5]. Total RNA was extracted from developmental stages (~ 35–40 mg), exposed larvae (~ 600 larvae) and spat (100–600 spat depending on metamorphosis success) using TRI Reagent RNA Isolation Reagent (Sigma-Aldrich) following the manufacturer’s protocol. The amount of cDNA template was 0.5 μg in 2018 experiments and 1 μg in 2019 experiments. Optimal primer efficiency for *CgNOS* was reached at an annealing temperature of 62 °C and a final primer concentration of 0.2 μM.

### DAF-FM diacetate assay

For NO detection in competent Pacific oyster larvae, eyed pediveliger larvae (derived from two males and two females) were obtained from the Roem van Yerseke hatchery in Yerseke, the Netherlands, and held at 23 °C in 100 L static tanks at the Ghent University in UV treated FSW with salinity and pH of 33.5 ± 0.5 ppt and pH 8.1 ± 0.1, respectively. Larvae were fed daily after daily water change with a microalgal mixture (1:1:2) of *T. lutea*, *C. muelleri* and *P. lutheri.*

Nitric oxide in oyster larvae was detected using the 4-amino-5-methylamino-2′,7′- difluorofluorescein (DAF-FM) diacetate, a non-fluorescent, cell-permeant compound that after hydrolysis to DAF-FM, reacts with NO to produce a relatively impermeable fluorescent triazolofluorescein derivative. Twenty to fifty competent larvae (24–26 dpf, 290–310 μm) were placed a 12 well plate in a total volume of 2.5 ml FSW, untreated or exposed to EPI, MK-801, SMIS and 7NI at 10^− 4^ M or AGH at 10^− 3^ M for 3 h, chemicals were then removed and 5 ml of FSW with microalgal feed was added to each well. Stock solution of 5 mM DAF-FM diacetate (Sigma Aldrich) was prepared with DMSO (Sigma-Aldrich) a few days prior to experiments and stored at − 20 °C until usage. After 1 h, water was changed again without microalgal feed and larvae were exposed to a final concentration of 7.5 μM DAF-FM diacetate in 2 ml FSW for 60 min in the dark. Spat from a previous day exposure to EPI were also incubated with 7.5 μM DAF-FM diacetate. Animals were washed twice with 1 ml FSW and incubated for 30 min in 2 ml FSW to allow complete de-esterification of intracellular diacetates. Animals were assessed alive using a Zeiss Axioskop 2 plus fluorescent microscope equipped with a FLUO filter set 38 (Ex: 470 ± 40 BP, Em: 525 ± 50 BP;) and pictures were taken using a monochrome Retiga3 Camera (QImaging) with Ocular V 2.0 Software (Digital Optics Limited) for imaging. To aid taking pictures of swimming larvae, animals were occasional anaesthetised with 7.5% MgCl_2_ in FSW. Animals not exposed to DAF-FM diacetate were also assessed, but exhibited only very weak fluorescence at the wavelength employed.

### In-situ hybridisation

Gene expression of *CgNOS* and *CgNR1* were localised in pediveliger larvae by in-situ hybridisation (ISH). Competent larvae, untreated 17 dpf and 3 hpe of EPI, as well as spat 24 hpe of EPI from the March 2019 spawning event were anaesthetised with 7.5% MgCl_2_ in FSW prior fixing in PaxGene Tissue system (PreAnalytix). Sectioning of larvae and execution of ISH followed the protocol previously described [5]. Riboprobes were produced from cDNA fragments from *CgNOS* (344 bp) and *CgNR1* (323 bp). Antisense riboprobes for DIG labelling were generated with specific forward primers and reverse primers with a T7 antisense extension for *CgNOS* (forward primer 5′-GGAATGTGGGACGTGTTGCC-3′, reverse primer + T7 extension 5′-TAATACGACTCACTATAGGGGCTCGGTCCTTCCACAGTGA-3′) and *CgNR1* (forward primer 5′-TGCAACTGGGACAAGAACGA-3′, reverse primer + T7 extension 5′- TAATACGACTCACTATAGGGTGCAACTGGGACAAGAACGA-3′) by PCR using MyTag PCR mix (Bioline) at 95 °C for 1 min, 35 cycles of 95 °C for 15 s, 65 °C (*CgNOS*) or 64 °C (*CgNR1*) for 15 s and 72 °C for 30 s with a final extension of 2 min at 72 °C. Sense riboprobes for *CgNOS* used as non-specific binding control in the ISH assay were amplified using the specific forward primer with a T7 sense extension and the reverse primer during the PCR. DIG labelling of all probes was conducted using the DIG RNA labelling kit (Roche) using an RNA T7 polymerase. After successful hybridisation, sections were mounted using a Vectamount (Vector Laboratories) and examined and photographed using an epi-fluorescent Arcturus XT Laser Capture Microdissection system (ThermoFisher Scientific) build on a Nikon Eclipse Ti-E microscope with triple-band DAPI-FITC-Texas Red excitation filter and a QImaging MicroPublisher Color RTV-5.0 CCD Camera (QImaging Corp.) for imaging.

Additional conventional hematoxylin and eosin (H&E) staining was performed for anatomy identification with the sections used for sense probes of *CgNOS* or *CgNR1* after removal of Vectamount. Unfortunately, some individuals on sections were lost during this procedure and consecutive H&E stained section were used instead.

### Immunofluorescent assay

For localisation of cGMP in larva, immunofluorescent assays were carried out using a rabbit anti-cGMP polyclonal antibody PAb (Sigma-Aldrich, 09–101) on sections of larvae and in whole mount. Before fixation larvae were relaxed with 7.5% MgCl_2_ and then fixed in PaxGene Tissue system (PreAnalytix).

Sections of competent larvae were prepared as for the ISH analysis previously described [5]. Sections were dewaxed in xylene for 2 × 5 min and gradually rehydrated through graded alcohol bath with 100% ethanol followed by 70% ethanol, each for 5 min, and a final 5 min bath in 1x PBS (0.02 M phosphate, 0.15 M NaCl, pH 7.1). Sections were washed in 1xPTA (PBS + 4% Triton X-100 (Sigma-Aldrich)) for 10 min and then pre-treated with blocking buffer (5% goat serum in 1xPTA) for 45 min in the dark. Blocking buffer was removed and sections were incubated overnight in the dark at 4 °C in blocking buffer with primary antibodies, either 1:500 anti-cGMP PAb or 1:200 rabbit anti-Salmonid MHC II PAb (Vertebrate Antibodies Ltd., UK) as rabbit antibody control for non-specific binding, or without a primary antibody functioning as control for non-specific secondary antibody control (Additional file 5). Sections were washed three times with 1xPTA for 10 min and then incubated for 2 h in the dark in blocking buffer with 1:100 secondary antibody Goat anti-Rabbit IgG (H + L) Cross-Adsorbed Secondary Antibody DyLight 550 (Invitrogen, SA5–10033). Slides were rinsed three times with 1xPTA for 10 min.

For whole-mount localisation of cGMP, larvae were decalcified in 10% EDTA in 1xPBS for 5 h, washed with 1xPBS for 5 min and 1xPTA for 10 min and then incubated for 24 h at 4 °C with blocking buffer. Next, larvae were incubated with primary antibody as above and incubated for 45–48 h in the dark at 4 °C with occasional shaking. Larvae were then washed three times over a period of 5 h in the dark on a shaking plate and then incubated for 21–24 h at 4 °C in the dark with the secondary antibody. After incubation, larvae were washed twice for 1 h in the dark on a rocking platform.

Section and individual whole larvae were mounted using Vectashield Antifade Mounting Medium with DAPI (Vector Laboratories) and were examined and photographed using an epi-fluorescent Arcturus XT Laser Capture Microdissection system (ThermoFisher Scientific) built on a Nikon Eclipse Ti-E microscope with G-2A filter for the DyLight 550 secondary antibody and UV-2A filter for the DAPI staining. An additional conventional H&E staining of a section was performed for anatomy identification after mounting medium was removed.

In addition to the ISH detection of *CgNOS*, an immunofluorescent assay was performed using a universal uNOS polyclonal antibody (PA1–38835, Invitrogen) at 1:100 and 1:200 dilutions in larval section and whole-mount larvae samples, but successful binding could not be obtained, not recommending this antibody for NOS detection in the Pacific oyster (Additional file 5).

A Western blot analysis was conducted to confirm successful binding of anti-cGMP PAb to oyster larvae protein. Thirty milligrams of 17 dpf oyster larvae were homogenized in 0.5 ml 1xPBS using a bead beater. The supernatant was aliquoted and protein concentration was quantified using a Pierce BCA Protein Assay Kit (ThermoScientific) following the manufacture’s protocol. Undiluted protein extracts were diluted 4:1 with 5x sodium dodecyl sulphate (SDS) sample buffer (0.5 M Tris–HCl, pH 6.8, 20% glycerol, 4% SDS, 0.2 M dithiothreitol, 0.02% bromophenol blue), boiled for 10 min and centrifuged for 2 min at 16,000 g. Fifteen microliter of prepared samples were loaded into a 10% Mini-PROTEAN TGX Precast Gel (Bio-Rad, UK) and subjected to SDS polyacrylamide gel electrophoresis (SDS-PAGE). Proteins were then electro-transferred to a nitro-cellulose membranes (Amersham Hybond ECL, GE Healthcare) using a Pierce G2 Fast blotter (ThermoScientific) for 7 min at 25 V. Membrane was blocked with 3% skimmed milk powder (SMP) in tris buffer saline (TBS, 0.02 M Trisma base, 0.5 M NaCl, pH 7.5) overnight at 4 °C. Membranes were then washed three times in TBST (TBS + 0.05% Teeen20) for 5 min and cut into strips. The membrane strips were individually incubated in 1:500 anti-cGMP PAB, 1:100 anti-uNOS PAB or without antibody (negative control) in TBS + 1% SMP for 2 h at RT and washed three times in TBST. Membranes were then incubated again with anti-rabbit IgG Peroxidase Conjugate (Sigma Aldrich) diluted 1:250 in TBS + 1% SMP for 1 h at RT. After a final wash with TBS positive bands showing antigen-PAb complexes in the membrane were developed with ImmPact DAB Peroxide Substrate Peroxidase (Vector Laboratories) following the manufacturer’s instructions.

### Data analysis

Metamorphosis success was calculated as percentages of larvae completing metamorphosis based on the total number of individuals in each vial presenting the average and standard error of three biological replicates per treatment. The non-parametric Kruskal–Wallis H-test was applied to analyse the effects between different treatments, followed by pairwise comparison using a generalisation of the Dunnett’s T3 method to trimmed means [90].

The gene expression patterns of all genes were analysed for three independent replicates for each sampling point using a one-way ANOVA follows by multiple pairwise comparisons using a Tukey’s Honestly Significant Difference Test. All statistical tests were run using the R software (R version 3.5.1) and the probability level of 0.05 was chosen as being significant.

## Supplementary Information

**Additional file 1.** Percentage metamorphosis in Pacific oyster larvae 19 dpf after 24 h continuous exposure to NO pathway inhibitors SMIS, AGH, 7-NI, L-NAME, L-NNA and ODQ.

**Additional file 2.** Percentage metamorphosis in Pacific oyster larvae after 3 h, 6 h and 14 h exposure to NO pathway inhibitors SMIS, AGH, 7-NI and ODQ.

**Additional file 3. **In-situ hybridisation of competent Pacific oyster larvae sections using digoxigenin labelled *CgNOS* sense riboprobes (non-specific binding controls) or no riboprobes (negative controls) for *CgNOS* and *CgNR1* subunit.

**Additional file 4.** cGMP immunostaining in whole-mount stained 14 dpf and 15 dpf larvae and sections of 16 dpf larvae.

**Additional file 5.** Immunostaining larvae and spat with anti-cGMP-PAb, anti-uNOS PAb, non-specific binding controls and negative controls for sections and whole-mount larvae as well as Western blot analysis.

## Data Availability

The datasets used and/or analysed during the current study are available from the corresponding author on reasonable request.

## Abbreviations

7-NI: 7-nitroindazole
AGH: aminoguanidine hemisulfate salt
ASO: apical sensory organ
cGMP: cyclic guanosine monophosphate
DAF-FM: 4-amino-5-methylamino-2′,7′-difluorofluorescein
dpf: days post fertilisation
EPI: epinephrine
ERK: extracellular-signal-regulated kinase
FSW: filtered fresh seawater
GTP: guanosine triphosphate
hpe: hours post exposure start
JNK: c-Jun NH2-terminal kinase
L-DOPA: levodopa
L-NAME: L-NG-nitroarginine methyl ester
L-NNA: L-NG-nitroarginine
MAPK: mitogen-activated protein kinase
NMDA: *N*-Methyl-D-aspartate
NO: nitric oxide
NOS: nitric oxide synthase
ODQ: 1H-[1,2,4]Oxadiazole[4,3-a]quinoxalin-1-one
PAb: polyclonal antibody
PDE: phosphodiesterase
PKG: protein kinases G
PSD-95: postsynaptic density proteins 95
sGC: soluble guanylyl cyclase
SIN-1: 3-morpholinosydnonimine chloride
SMIS: s-methylisothiourea hemisulfate salt
SNAP: S-nitroso-N-acetyl-penicillamine
SNP: sodium nitroprusside dihydrate
