# Supplementary material for: Bivalves are NO different: nitric oxide as negative regulator of metamorphosis in the Pacific oyster, Crassostrea gigas
Source: BMC Dev Biol. 2020 Nov 23;20:23. doi: 10.1186/s12861-020-00232-2 (PMC7686737; doi:10.1186/s12861-020-00232-2)

Additional file 1: Percentage (%) of metamorphosis in Pacific oyster larvae after 24 h continuous exposure to single treatments (black bars) of NO pathways inhibitors SMIS, AGH, 7-NI, L-NAME, L-NNA and ODQ, at different concentrations, as well as known inducers epinephrine (EPI; light grey bars) and MK-801 (MK; grey bars) at 10^{-4} M for 3 h, a DMSO (black-stripe bars) and a no treatment control (open bars). Data were collected 24 h post exposure start. *C. gigas* larvae from December 2018 experiment with competent larvae 19 dpf were used for all treatments except for ODQ, for which 18 dpf larvae were used. Error bars represent standard error. Different lower-case letters represent significant differences with $p<0.05$.

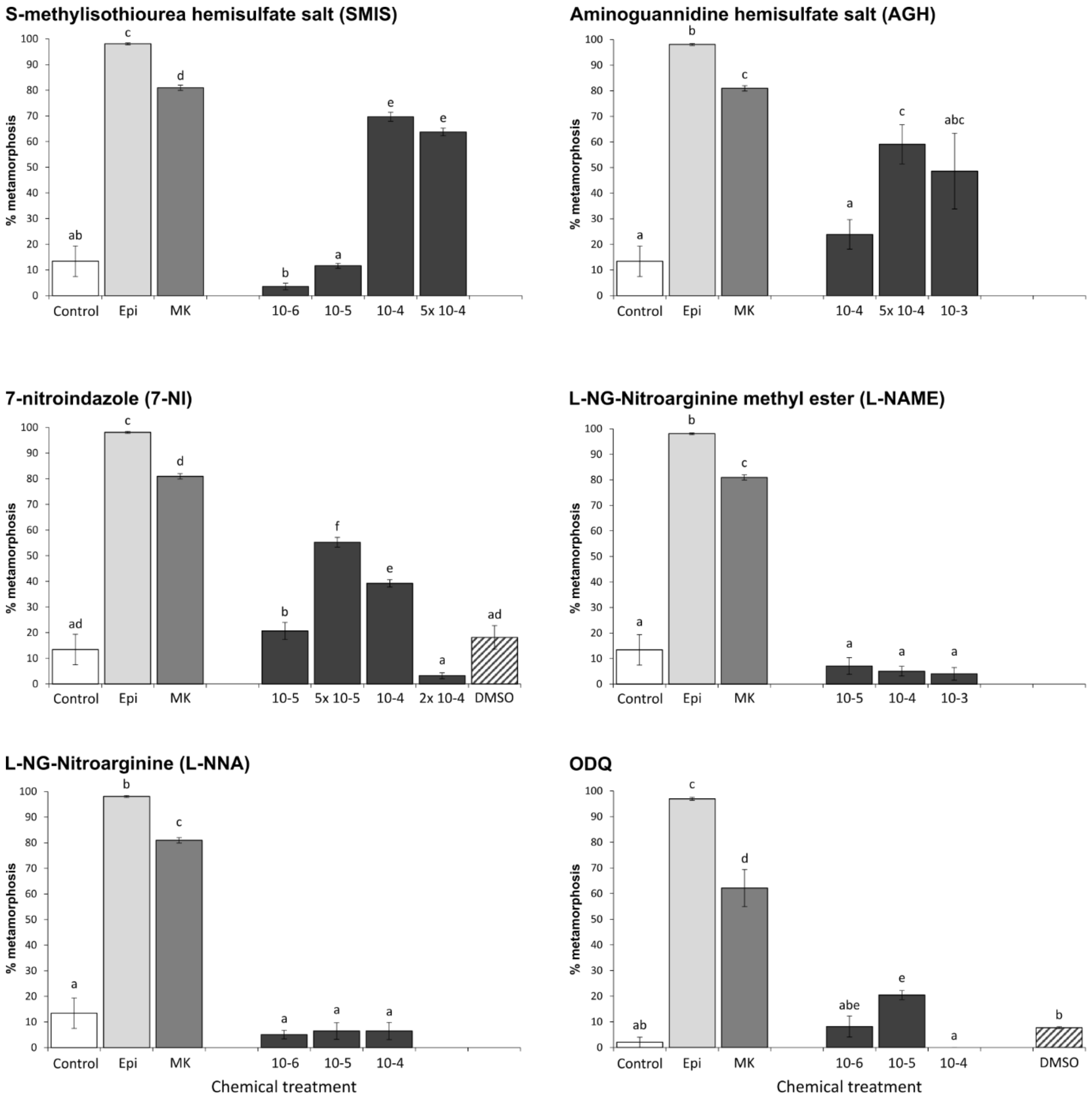

Supplement: Supplementary file 1 — Additional file 1. Percentage metamorphosis in Pacific oyster larvae 19 dpf after 24 h continuous exposure to NO pathway inhibitors SMIS, AGH, 7-NI, L-NAME, L-NNA and ODQ. [file 12861_2020_232_MOESM1_ESM.pdf]
