# Supplementary material for: Bivalves are NO different: nitric oxide as negative regulator of metamorphosis in the Pacific oyster, Crassostrea gigas
Source: BMC Dev Biol. 2020 Nov 23;20:23. doi: 10.1186/s12861-020-00232-2 (PMC7686737; doi:10.1186/s12861-020-00232-2)

Additional file 2: Percentage (%) of metamorphosis in Pacific oyster larvae after 3 h, 6h or 24 h continuous exposure to single treatments (black bars) of NO pathways inhibitors SMIS, AGH, 7-NI and ODQ at different concentrations, as well as known inducers epinephrine (EPI; light grey bars) and MK-801 (MK; grey bars) at 10^{-4} M for 3 h, a DMSO (black-stripe bars) and a non-treatment control (open bars). Data were collected 24 h post exposure start. *C. gigas* larvae from March 2019 experiment with competent larvae 17 dpf and 18 dpf were used. Error bars represent standard error. Different lower-case letters represent significant differences with $p<0.05$.

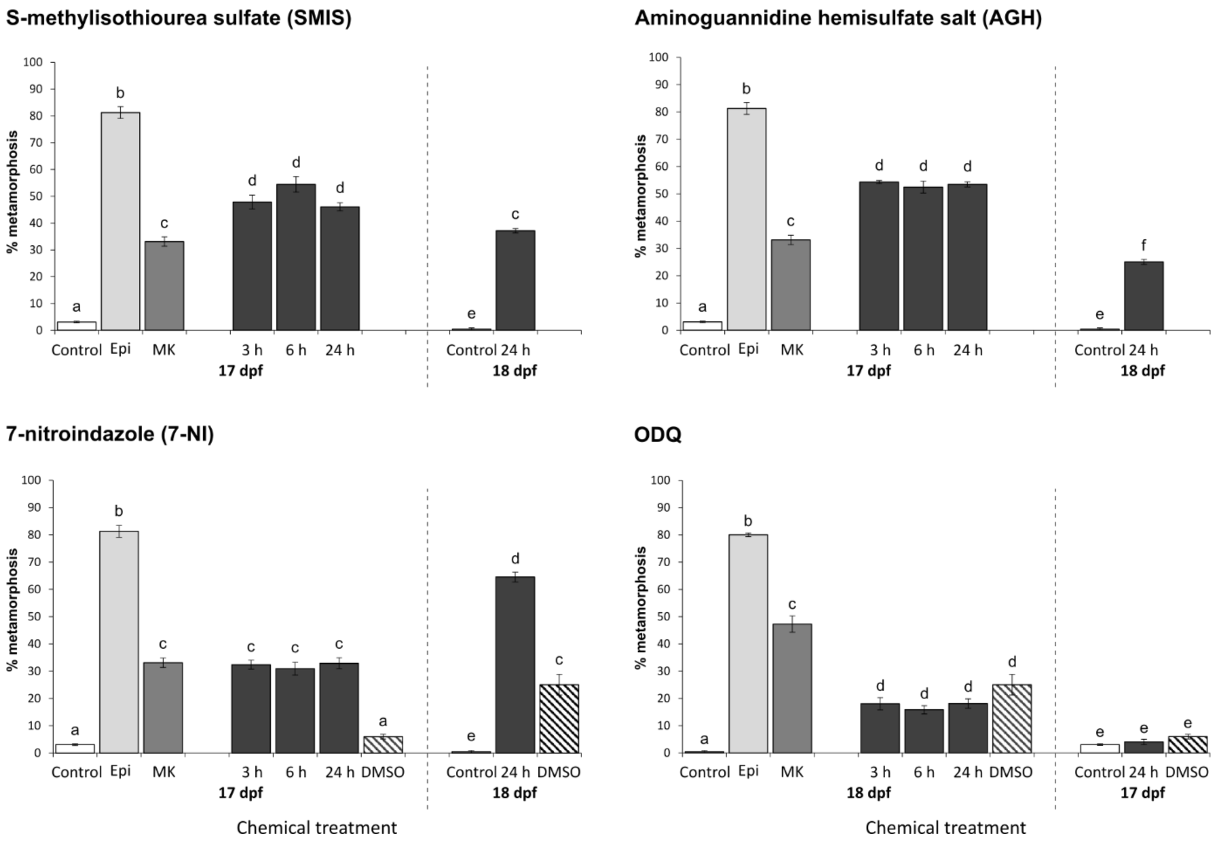

Supplement: Supplementary file 2 — Additional file 2. Percentage metamorphosis in Pacific oyster larvae after 3 h, 6 h and 14 h exposure to NO pathway inhibitors SMIS, AGH, 7-NI and ODQ. [file 12861_2020_232_MOESM2_ESM.pdf]
