# Supplementary material for: Bivalves are NO different: nitric oxide as negative regulator of metamorphosis in the Pacific oyster, Crassostrea gigas
Source: BMC Dev Biol. 2020 Nov 23;20:23. doi: 10.1186/s12861-020-00232-2 (PMC7686737; doi:10.1186/s12861-020-00232-2)

Additional file 3: *In-situ* hybridisation of competent Pacific oyster larvae sections using digoxigenin labelled *CgNOS* sense riboprobes (non-specific binding controls) or no riboprobes (negative controls) for *CgNOS* and *CgNR1* subunit. A -C) Frontal sections of foot area, (D-E) Transverse section of larvae, and (F) Sagittal section whole spat. *: Fast red dye unspecific binding mostly in remains of periostracum. n.a.: not available. Scale bar: 50 µm.

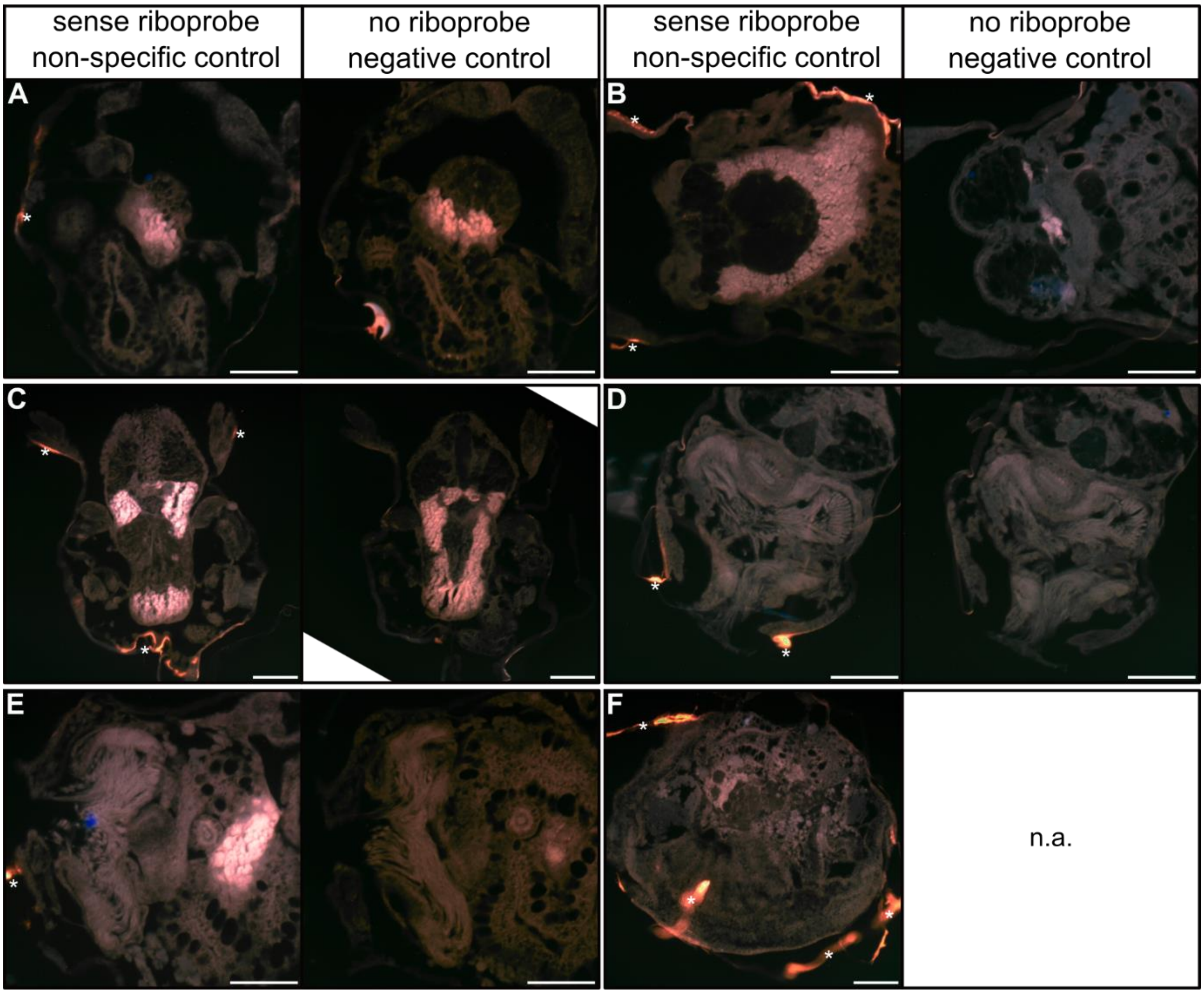

Supplement: Supplementary file 3 — Additional file 3. In-situ hybridisation of competent Pacific oyster larvae sections using digoxigenin labelled CgNOS sense riboprobes (non-specific binding controls) or no riboprobes (negative controls) for CgNOS and CgNR1 subunit. [file 12861_2020_232_MOESM3_ESM.pdf]
