# Supplementary material for: Bivalves are NO different: nitric oxide as negative regulator of metamorphosis in the Pacific oyster, Crassostrea gigas
Source: BMC Dev Biol. 2020 Nov 23;20:23. doi: 10.1186/s12861-020-00232-2 (PMC7686737; doi:10.1186/s12861-020-00232-2)

Additional file 4: cGMP immunostaining in Pacific oyster larvae of whole-mount stained 14 dpf larva (A) and 15 dpf larva (B), both flattened with cover slip, as well as sagittal section of 16 dpf larva (C) with anti-cGMP PAb, anti-salmonid MHC II PAb as non-specific binding control, and without primary antibody as negative control for consecutive sections accompanied with superimposed DAPI/cGMP signals and/or H&E staining. Scale bar: 50 µm.

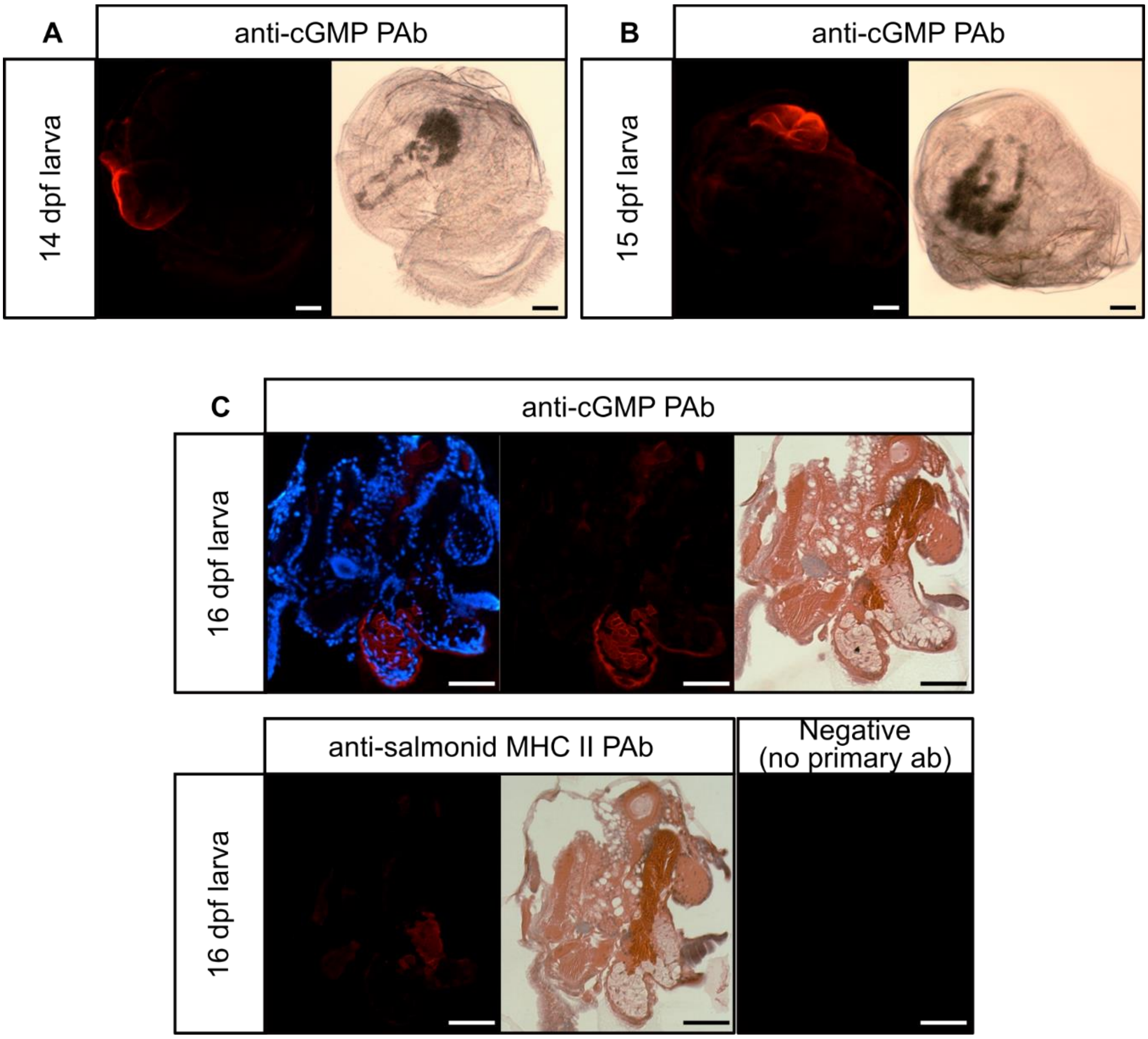

Supplement: Supplementary file 4 — Additional file 4. cGMP immunostaining in whole-mount stained 14 dpf and 15 dpf larvae and sections of 16 dpf larvae. [file 12861_2020_232_MOESM4_ESM.pdf]
