# Supplementary material for: Bivalves are NO different: nitric oxide as negative regulator of metamorphosis in the Pacific oyster, Crassostrea gigas
Source: BMC Dev Biol. 2020 Nov 23;20:23. doi: 10.1186/s12861-020-00232-2 (PMC7686737; doi:10.1186/s12861-020-00232-2)
Supplement: Supplementary file 5 — Additional file 5. Immunostaining larvae and spat with anti-cGMP-PAb, anti-uNOS PAb, non-specific binding controls and negative controls for sections and whole-mount larvae as well as Western blot analysis. [file 12861_2020_232_MOESM5_ESM.pdf]

Additional file 5: Immunostaining in Pacific oyster larvae spat with anti-cGMP PAb, anti-salmonid MHC II PAb as non-specific binding control, and without primary antibody as negative control for (A) consecutive sections presented in Figure 6 and representative for (B) whole-mount staining of larvae and spat with non-specific binding control and negative control. Immunofluorescent images for anti-salmonid MHC II PAb are accompanied with H&E staining of the same section (except for 17 dpf larva 6 hpe, which was lost during processing and replaced with DAPI stained image of same section for histology structure identification). (C) Immunostaining with anti-uNOS PAB (PA1-38835, Invitrogen) in larval sections and whole-mount staining (flattened with cover slip) accompanied with DAPI staining. No successful binding was obtained and we do not recommend this antibody for NOS detection in the Pacific oyster. (D) Western blot for anti-cGMP, anti-uNOS and no antibody (negative control NEG) with two independent blots perform from two independent oyster protein extracts. Scale bar: 50 µm.

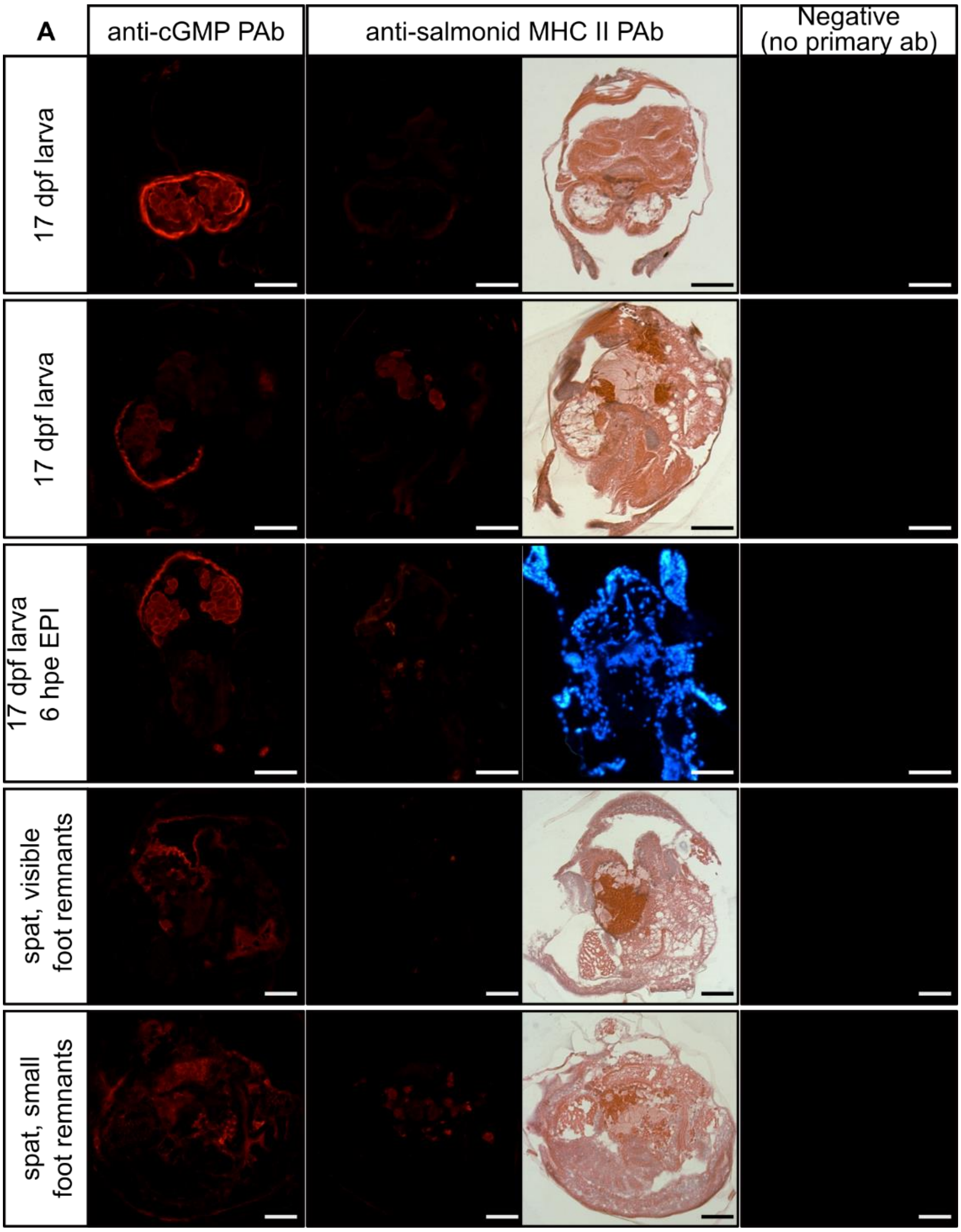

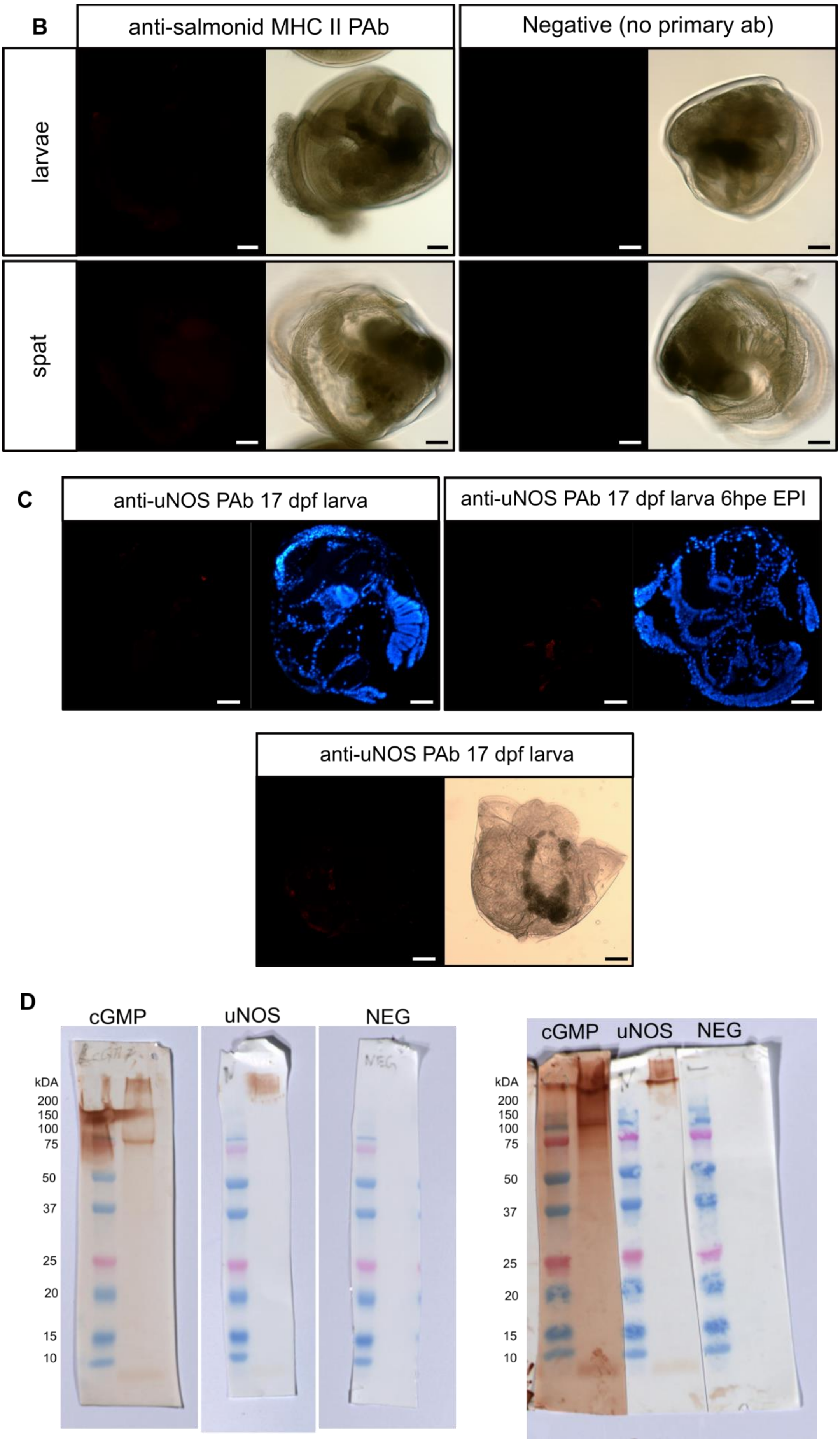
B
anti-salmonid MHC II PAb
Negative (no primary ab)
larvae
spat
C
anti-uNOS PAb 17 dpf larva
anti-uNOS PAb 17 dpf larva 6hpe EPI
anti-uNOS PAb 17 dpf larva
D
cGMP
uNOS
NEG
kDA
200
150
100
75
50
37
25
20
15
10
cGMP
uNOS
NEG
kDA
200
150
100
75
50
37
25
20
15
10
